# Low-cost portable microwave sensor for non-invasive monitoring of blood glucose level: novel design utilizing a four-cell CSRR hexagonal configuration

**DOI:** 10.1038/s41598-020-72114-3

**Published:** 2020-09-16

**Authors:** Ala Eldin Omer, George Shaker, Safieddin Safavi-Naeini, Hamid Kokabi, Georges Alquié, Frédérique Deshours, Raed M. Shubair

**Affiliations:** 1 Department of Electrical and Computer Engineering, Centre for Intelligent Antenna and Radio Systems (CIARS), University of Waterloo, Waterloo, ON Canada; 2 Group of Electrical Engineering Paris (GeePs), UMR CNRS-CentraleSupelec – University Paris-Saclay – Sorbonne University, Paris, France; 3 Department of Electrical Engineering and Computer Science, Massachusetts Institute of Technology (MIT), Cambridge, MA USA; 4 Department of Electrical and Computer Engineering, New York University Abu Dhabi, Abu Dhabi, UAE

**Keywords:** Biotechnology, Health care, Engineering, Endocrine system and metabolic diseases

## Abstract

This article presents a novel design of portable planar microwave sensor for fast, accurate, and non-invasive monitoring of the blood glucose level as an effective technique for diabetes control and prevention. The proposed sensor design incorporates four cells of hexagonal-shaped complementary split ring resonators (CSRRs), arranged in a honey-cell configuration, and fabricated on a thin sheet of an FR4 dielectric substrate.The CSRR sensing elements are coupled via a planar microstrip-line to a radar board operating in the ISM band 2.4–2.5 GHz. The integrated sensor shows an impressive detection capability and a remarkable sensitivity of blood glucose levels (BGLs). The superior detection capability is attributed to the enhanced design of the CSRR sensing elements that expose the glucose samples to an intense interaction with the electromagnetic fields highly concentrated around the sensing region at the induced resonances. This feature enables the developed sensor to detect extremely delicate variations in the electromagnetic properties that characterize the varying-level glucose samples. The desired performance of the fabricated sensor is practically validated through in-vitro measurements using a convenient setup of Vector Network Analyzer (VNA) that records notable traces of frequency-shift responses when the sensor is loaded with samples of 70–120 mg/dL glucose concentrations. This is also demonstrated in the radar-driven prototype where the raw data collected at the radar receiving channel shows obvious patterns that reflect glucose-level variations. Furthermore, the differences in the sensor responses for tested glucose samples are quantified by applying the Principal Component Analysis (PCA) machine learning algorithm. The proposed sensor, beside its impressive detection capability of the diabetes-spectrum glucose levels, has several other favorable attributes including compact size, simple fabrication, affordable cost, non-ionizing nature, and minimum health risk or impact. Such attractive features promote the proposed sensor as a possible candidate for non-invasive glucose levels monitoring for diabetes as evidenced by the preliminary results from a proof-of-concept in-vivo experiment of tracking an individual’s BGL by placing his fingertip onto the sensor. The presented system is a developmental platform towards radar-driven wearable continuous BGL monitors.

## Introduction

Diabetes is a metabolic disorder that is caused by the malfunction of steady hormone insulin production by the pancreas, thus degrading the cell’s ability to absorb glucose from the bloodstream^1^. Diabetes is addressed based on the glucose level which cannot be physiologically maintained and standardized for the diabetic patient within the normal desired range, thus indicating the need for the patient to undergo regular medical inspection^2,3^. Typical glucose levels in human beings’ blood for Type-2 diabetes—which is widespread among 90% of the diabetics’ community—normally vary in the range from 72 to 126 mg/dL before having a meal and should be less than 153 mg/dL within 1–2 h after a meal^4^. Negligence to preserve certain glycemic targets would probably put the patient at the risk of experiencing the extreme events of hyperglycemia resulting into serious health complications such as heart attack, stroke, kidney failure, blindness, amputation, etc.^1^. Alternatively, for very low glycemia levels, patients would encounter hypoglycemia that could rapidly lead to life-threating events such as coma or death^1^. These drastic effects of diabetes could be averted by having an early diagnosis and following a treatment plan that involves renovated lifestyle, continuous glucose monitoring (CGM), and regular medical care with the proper medication.

Invasive and minimally invasive methods have been widely used in the market to check for blood glucose levels. Invasive devices estimate the glucose following a finger-pricking procedure to analyze a blood sample from the fingertip on a test strip. However, the pain, expenses, risk for infection, and inconvenience associated with such self-monitoring technology can lead to patient noncompliance and insufficient number of daily measurements^5^. On the other hand, currently available CGMs are described to be minimally invasive since the blood vessels are not actually punctured but rather a sensor is put into contact with the interstitial fluid (ISF) subcutaneously by pushing a tiny needle into the arm or abdominal wall. So, these sensors estimate the glucose level in ISF every few minutes via measuring the current flowing through a very thin wire to the external transmitter^6^. This current is produced proportionally to the amount of glucose being oxidized in a chemical reaction in the presence of specific enzymes (e.g. enzyme glucose oxidase). A transmitter is attached external to the implanted sensor to send the measurements frequently via wireless signals to a receiver that processes the data and displays the glucose readings. Despite being quite valuable, most of the current minimally invasive CGMs have issues either in accuracy or sustainability, where most of these CGM devices are implanted inside the body, and hence a replacement is recommended to avoid any side effects on the long term. Additionally, the total expense for the end users is relatively high and sometimes is not affordable on a frequent basis^6^.

Accordingly, there have been a demand for non-invasive pain-free glucose monitors that would guarantee more affordable, careful and comfortable control of BGLs with no replacement over the long term^7^. Significant number of researchers have been investigating the development of more innovative non-invasive (NI) glucose detection systems^7^. Most of these systems are based on optical methods where the scattering of light at several wavelengths is used to detect the glucose concentrations by measuring some optical parameters such as Raman spectroscopy^8^, optical coherence tomography (OCT)^9^, photoacoustic^10^, impedance^11^, and near infra-red (NIR) spectroscopy^12^. However, massive, power-hungry, and expensive instruments are commonly required to conduct these optical measurements at short wavelengths. In addition, these techniques are known to have low signal-to-noise ratio (SNR) and large errors inherent from the calibration drift, thermal noise, and vulnerability to physiological (e.g. body temperature) and environmental (e.g. humidity) conditions^13^. The accessible biological fluids such as tears^14–16^, saliva^17^, sweat^18,19^, and breath condensation^20^, have been utilized in numerous enzyme-based electrochemical techniques to correlate their glucose content to the glycemia levels. Despite being quite innovative, a lagging correlation is shown to the physical BGL variations, in addition to their susceptibility to metabolic changes. Likewise, the transdermal techniques where reverse iontophoresis methods are used to extract and measure the BGL from ISF, are shown to be very expensive, deteriorating over time, and uncomfortable for injecting electrical current through the skin^21,22^. Several devices have recently been developed using the aforementioned techniques with promising preliminary results. Table 1 shows some of these devices and their varying development status as a proof-of-concept, or waiting for regularity approval, or even been it to the market while continuing to improve the technology^6^. Many other devices were released earlier but did not last long in the market for some difficulties. For instance, GlucoWatch based on the reverse iontophoresis technique, was approved by FDA for its reasonable accuracy in clinical and home trials, yet it was dropped due to some issues in reliability and consistency. Another example is Pendra (based on impedance spectroscopy) was CE approved and marketed for short period but dropped later for its poor accuracy. Some other devices did not initially reach out to the market for lacking funds to finalize their designs (e.g. C8 Medisensors based on Raman spectroscopy)^6^.

**Table 1:** Non-invasive glucose monitoring devices currently available in the market or close to release.

Device	Technology	Placement	Type	Status
Combo Glucometer (Cnoga Medical)^23^	NIR spectroscopy	Finger	NI NCGM	Available
NBM-200G (OrSense)^24^	NIR spectroscopy	Finger	NI CGM	Dropped
HELO Extense (World Global Network)^25^	NIR spectroscopy	Finger	NI NCGM	Available
GlucoTrack (Integrity Applications)^26^	Ultrasound, thermal, electromagnetic sensing	Ear lobe	NI NCGM	Approved and commercialized in Europe
GlucoSense	Optical/laser technology	Finger	NI NCGM	Performed one clinical trial. Aiming for more after further development
SugarBEAT (Nemaura Medical)^27^	Reverse iontophoresis (Electric current)	Upper Arm	NI CGM	Waiting for CE approval
Wizmi (Wear2b Ltd)^28^	NIR spectroscopy	Arm wrist	NI CGM	Proof of concept

Radio frequency (RF)/microwave sensing techniques have shown to be more promising for non-invasive glucose monitoring due to the non-destructive nature of the electromagnetic (EM) waves when penetrating inside the biological tissues compared to other ionizing types of radiation such as X-rays that bring hazardous effects. A variety of reflection and/or transmission methods using antennas^29^ or waveguides^30^ while relying on costly and bulky VNAs^31^ have been explored to characterize the EM properties (i.e. dielectric constant and loss) of blood samples in different setups. Remarkably, a distinct correlation has been demonstrated between the measured blood properties and its glucose content. However, most of these RF systems are not practicable for immediate in-house usage by diabetics due to their cost, complexity, size, and weight. Researchers have also been exploring sensing structures in the millimeter(mm)-wave band that enables shorter wavelengths and relatively acceptable penetration depth adequate to interrogate the glucose concentrations in body regions with thin tissues. For instance, in another work^32,33^ we proposed a miniature sensing system that utilized a 60 GHz mm-wave radar for monitoring the blood glucose level. As a proof-of-concept the system was able to distinguish different synthetic blood samples of clinically diabetes-relevant concentrations in the range of 50–350 mg/dL by analyzing the backscattered signals from the sample-filled test tube placed at a distance from a compact radar sensor. The technique has proved the efficacy of blood glucose detection at mm-wave frequencies as a new concept and now is under development for further improvement to enable continuous glucose monitoring in a wearable format. The mm-wave spectrum has also been used by the authors in^29^, where a pair of microstrip patch antennas operating at 60 GHz were used to monitor the varying concentrations of glucose solutions inside acrylic tank placed in-between the two antennas by using the magnitude of the transmission coefficient S_21_ as a sensing parameter. A very low sensitivity of about 0.25 dB change in |S_21_| was reported for each 1,000 mg/dL change in glucose concentration. The system has shown poor sensitivity results but with reasonable correlation when tested in a controlled environment on ten male subjects of non-diabetes condition. The authors are progressively working in this sensor to have it as a hand-held device (named GlucoWise) that takes glucose readings on the earlobe or the skin between the thumb and forefinger.

Out of the many RF/microwave characterization techniques, the resonant-based methods are more favored and attractive to acquire high precision and accuracy in the sensing applications. They feature instant measurement of the dielectric permittivity of liquids; using simple, low-power and cost-effective structures^34^. They principally work based on tracking the resonance characteristics (resonant frequency, amplitude, width or quality coefficient) for permittivity changes induced by glucose variations in the tissue. For instance, microstrip open-loop resonators were used in^35^ to characterize the dielectric properties of small volumes (up to 25 µL) of glucose aqueous solutions in the frequency range 2–7 GHz using the quality factor as the sensing parameter. However, the glucose concentrations (1,250–10,000 mg/dL) considered in the study were very large and irrelevant to diabetes. Many researchers have recently explored diverse applications for the resonant planar circuits after incorporating the artificially-engineered materials “metamaterials”^36,37^. Integrating these materials of unique EM properties in the microwave/RF circuits helps realizing compact miniaturized systems of low cost, higher efficiency and sensitivity for non-invasive BGL monitoring. In particular, microwave sensors would significantly improve their sensitivity for characterizing the glucose samples under test (SUT) when metamaterials are incorporated for creating strong electric and magnetic field localization in the integrated sensing structure. Examples of such microwave sensors that couple the metamaterial units to the microstrip excitation-line are the split-ring resonators (SRR)^38,39^, complementary SRRs (CSRRs)^40^, and complementary electric-LC resonator^41^. The sensor in^38^ integrated two circular-shaped split-ring resonators for sensing the sample glucose level. One resonator was used for glucose sensing and the other as a reference to calibrate-out the temperature effects. The study in^40^ used a ring-and-horn complementary structure of 2.074 GHz resonance frequency to detect the glucose concentrations in the range 2,000–10,000 mg/dL. Another metamaterial resonator operating at 3–5 GHz was used in^42^ to detect the glucose concentrations 68–150 mg/dL in small droplets of human serums. A recent study proposed a closed-loop enclosed split-ring resonator operating in the frequency band 2–5 GHz to sense aqueous glucose concentrations in the range 50–400 mg/dL at a sensitivity of about 82 MHz/(mg/ml) in the reflection coefficient S_11_ response^43^. However, most of the published resonance-based sensors lack the frequency resolution required to capture the practical range of diabetes glucose levels. In fact, most of the earlier works have very low sensitivity and cannot accurately measure the glucose concentration levels seen during normal, hypoglycaemia and hyperglycemia in diabetes patients. Additionally, they used quite complex and sophisticated technologies for investigating the dielectric properties of blood.

**Figure 1:**
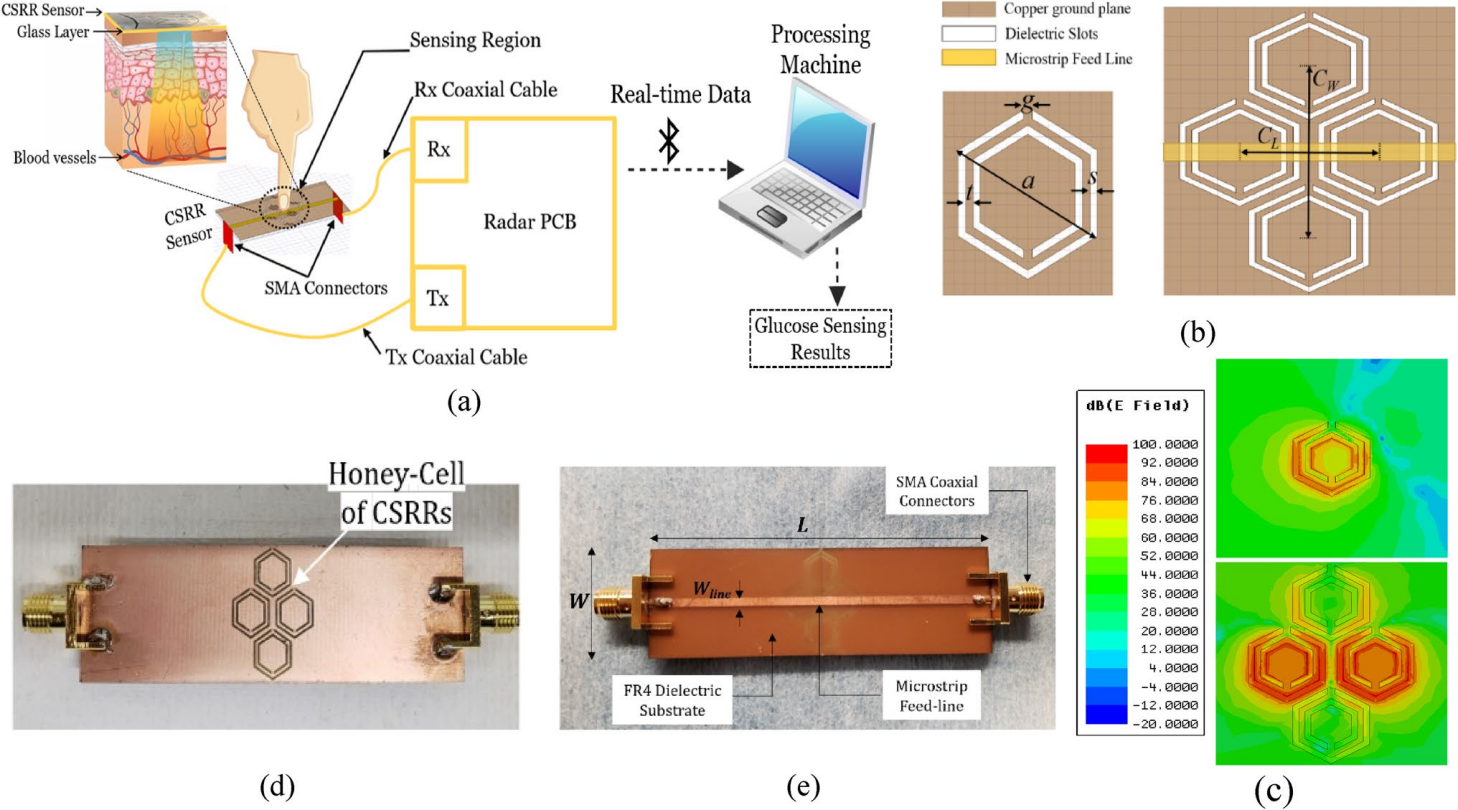
The proposed honey-cell CSRR sensor (**a**) General conceptual illustration of the portable radar-driven sensor for measuring the glucose level non-invasively from the fingertip by sending electromagnetic waves of small wavelength into the blood vessels, (**b**) configuration of the CSRRs sensing elements in the ground copper plane (top view). The topology of the hexagonal unit-cell with the geometrical parameters is also shown, (**c**) electric field distribution on the CSRR surface at 3.0 GHz resonant frequency for the improved and conventional topologies. The sketch portrays an intense electric field coupled to the CSRR area with higher magnitudes around the dielectric slits and the routes in-between. Therefore, this area is identified as the sensing region for the glucose samples to acquire strong interaction with the coupled near-field, and therefore induce notable variations in the intrinsic characteristics of the sensor in response to subtle variations in EM properties of different glucose concentrations. The honey-cell design exhibits higher electric field localization with an intensity up to 10^5^ V/m over the sensing region compared to that of a single hexagonal cell, (**d**) fabricated prototype of the sensor with the top view showing the ground plane where the honey-cell CSRRs are patterned, and (**e**) bottom view showing the microstrip-line used to excite the CSRRs.

Herein, we propose a portable prototype of an improved microwave sensor integrated with a low-cost 2.45 GHz ISM band radar for measuring the glucose level non-invasively in the blood tissue as illustrated in Fig. 1a. The sensor element is realized using a microstrip structure with an improved CSRRs configuration engraved in copper ground plane in a honey-cell fashion as shown in Fig. 1b. This honey-cell design is favored to alleviate the sensitivity limitations of the conventional SRR/CSRR structures by inducing an intensified electric field over a large sensing region adequate for fingertip or any fixture placement. Figure 1c, sketches the corresponding electric field distribution on the honey-cell CSRR sensing surface at 3.0 GHz resonant frequency (prior-loading) and compares it against that of a single hexagonal CSRR. It is clearly observed the intense electric field coupled to the CSRRs area (14 × 19 mm^2^) with higher magnitudes of about 10^5^ V/m around the dielectric slits and the routes in-between. Having most of the energy accommodated by the CSRR meta-elements is beneficial to enhance the sensitivity for characterizing the tiny variations in the dielectric properties of the targeted glucose concentrations when loaded onto the CSRRs surface. The primary objective of the proposed biosensor is to non-invasively monitor the BGL changes for diabetics. Towards that goal, the sensor is primarily experimented to detect the change in the glucose levels in blood mimicking solutions of concentrations in the range of Type-2 diabetes. This is done by analyzing the sensor transmission signals as reflected on the radar receiving port using signal processing and machine learning techniques to identify different glucose levels. The detection capability of the sensor to the varying glucose levels is also demonstrated by the frequency shifts in the reflection and transmission resonances when tested using a conventional VNA setup. Different topologies of the CSRRs have been studied using experimental and numerical analysis to compare their sensitivity performance. The compact-layout of the honey-cell sensor has demonstrated greater resonant frequency deviation in response to changes in glucose levels of interest. It scores an outstanding glucose sensitivity with frequency resolution of about 94 MHz/(mg/ml) outperforming other state-of-the-art microwave glucose sensors. Realizing larger frequency shifts (higher resolution) enables our sensor to be more robust against additive noise and other measurement uncertainties. Additionally, the sensor would be able to achieve more precise, easy and straightforward readings when integrated with any readout circuitry of wide dynamic ranges^44^. The improved CSRR sensor is capable of reproducibly detecting glucose concentrations ranging from 0 to 300 mg/dL in physiological solutions designed to mimic the practical diabetic condition with the precision of ~ 5 mg/dL which should provide sufficient sensitivity for accurate real-time monitoring of glycemia levels in patients with diabetes. The integrated honey-cell sensor has the advantages of being portable, non-invasive, low-cost, and simply-fabricated in an electrically small size. This work is a fundamental step towards developing the sensor for intermittent glucose levels monitoring by placing the fingertip along the sensing area as illustrated in Fig. 1a. The current design could also be adapted as a wearable device for monitoring glucose levels in diabetics by fabricating the CSRR sensing elements on a flexible substrate while being energized by an appropriate electronic reader.

## Results and discussion

In this section the proposed microwave glucose sensor is presented alongside with a systematic design approach, featured design parameters, dependency of resonance characteristics, numerical analysis, glucose sensing mechanism and the various accomplished in-vitro and in-vivo experiments for glucose concentration measurements. A comprehensive discussion including the influence of different geometrical parameters on the resonance profile, the sensitivity analysis for glucose detection under different topologies, and the signal processing and machine learning algorithms used to intelligently analyze the sensor’s raw data are also provided.

### CSRR-based sensor design

The proposed biosensor is numerically designed to operate around 2.45 GHz when used for glucose detection. This is chosen to match the Industrial, Scientific, and Medical (ISM) band 2.4–2.5 GHz when the sensor is integrated with the radar system^45^. In fact, operating in this range of frequency would also allow adequate penetration depth for the induced EM waves to reach the blood vessels in the fingertip. This penetration is to a certain extent maximized in our sensor given the concentrated energy of the sensing region where the fingertip is placed^46^. Additionally, at this frequency there is a higher possibility to identify different glucose concentrations of interest due to the small loss introduced (*tanδ* ~ 0*.*13). Therefore, sensing at this frequency would probably bring a considerable sensitivity despite the small changes in dielectric properties from one glucose level to another^47^. These variations in dielectric constant and loss tangent are studied for the desired concentrations across the centimeter-band 1–6 GHz using the first order Debye relaxation model proposed in^48^ for glucose aquatic solutions. A slight increase in ${{\epsilon }_{r}}^{^{\prime}}$ and, conversely, a tenuous decrease in *tanδ* are observed with an increased glucose concentration. In this tendency, it is also observed that the percent variation in *tanδ* is much higher than its corresponding on ${{\epsilon }_{r}}^{^{\prime}}$ (e.g. for a 10 mg/dL change at 2.4 GHz, the percent change in *tanδ* is about 0.4% while the variance in ${{\epsilon }_{r}}^{^{\prime}}$ is approximately 0.005%).

The sensor structure is primarily inspired from the metamaterial technology by incorporating four similar cells of hexagonal-shaped complementary split-ring resonators (CSRRs) with localized elements in a novel configuration as shown in Fig. 1b. The four CSRRs are configured in a honey-cell pattern and engraved at depth 35 µm in the copper ground plane of an FR4 dielectric PCB (Fig. 1d) of dielectric permittivity ${\epsilon }_{r}^{^{\prime}}$ = 4.4, loss tangent $tan\delta$ = 0.02, length *L* = 66 mm, width *W* = 20 mm, and thickness *h* = 0*.*8 mm*.* The magnetic walls of the passive CSRRs are oriented perpendicularly to a planar microstrip-line (MTL) etched on the upper face of the substrate as shown in Fig. 1e. This alignment would guarantee an electric excitation for the resonance with time-varying electric field between the MTL strip and the ground plane. The dimensions of the feed line are optimized to 66 × 1.5 mm^2^ in order to obtain a 50 Ω characteristic impedance that perfectly matches the SMA (SubMiniature version A) connectors of the driving power source. Two topologies for the honey-cell configuration are realized, compact and dispersed. Accordingly, two hexagonal cells are placed horizontally along the axis of the MTL strip and detached with distance *C*_*L*_ = 7*.*6 mm (compact) and *C*_*L*_ = 12*.*6 mm (dispersed) between their corresponding geometric centres. The honey-cell design is completed by setting the other two hexagonal cells in a vertical placement with *C*_*W*_ = 12 mm between their corresponding centres. As sketched in Fig. 1b, the unit-cell of each CSRR is composed of two concentric rings (outer and inner) of hexagonal shape nested within each other with a coupling-gap *t* = 0*.*4 mm in between. The loop of each ring has a dielectric slit of side-width *s* = 0*.*4 mm and ends in a metallic slot of width *g* = 0*.*4 mm*.* The diagonal length of the outer ring is *a* = 7*.*6 mm, while that of the inner one is set to *b* = 6*.*0 mm. The split gaps for both rings are on diametrically opposite sides of each other.

**Figure 2:**
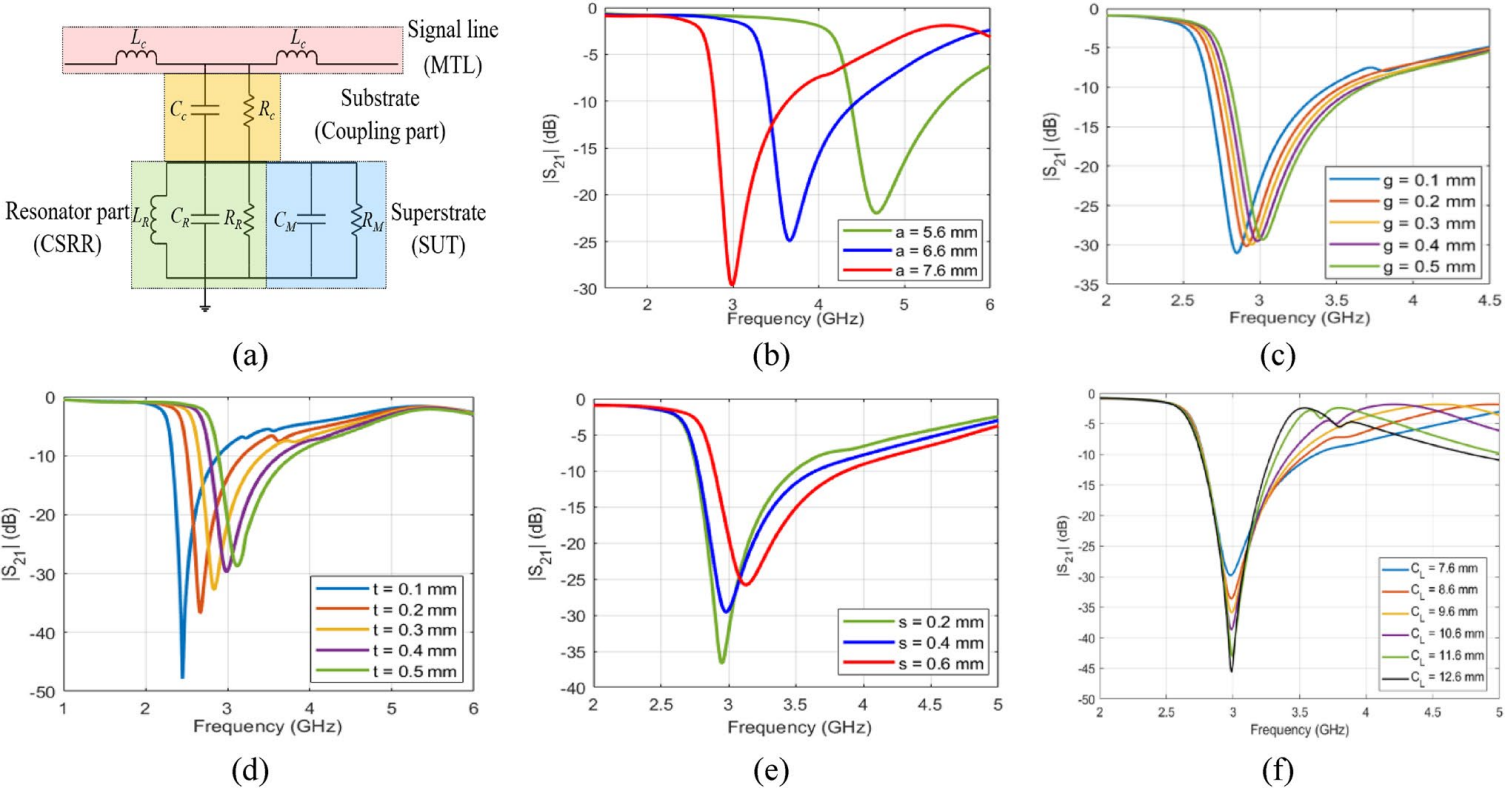
(**a**) Equivalent electrical model of the CSRR sensor under loading condition. (**b**–**f**) Simulated transmission coefficient S_21_ of the unloaded honey-cell CSRR at different (**b**) diagonal length of *a* (**c**) split gap *g* (**d**) side width *s* (**e**) slots-coupling distance *t*, and (**f**) center-to-center coupling distance *C*_*L*_. The primary influence of each of these parameters on *f*_*R*_ is numerically investigated. Since the diagonal length *a* is proportional to the length of the dielectric slot, increasing *a* would directly increase the resultant *L*_*e*_ and therefore reducing *f*_*R*_ as shown in (**b**) for *a* changing from 5.6 to 7.6 mm. (**c**) depicts how *f*_*R*_ is shifted towards higher frequencies (i.e. increasing) as the split gap *g* is increasing from 0.1 to 0.5 mm. Similar functionality can be observed in (**d**) for the case of slots-coupling distance *t*. Moreover, *f*_*R*_ is also slightly increasing with an increased side-width *s* as shown in (**e**). Both behaviors can be explained by the fact that the side width, slots-coupling distance, and split gap are all inversely proportional to the effective capacitance *C*_*e*_, thereby increasing these parameters will probably increase *f*_*R*_. The effect of the center-to-center separation distance is studied in (**f**) that shows a higher magnitude for the transmission response (i.e. insertion loss), and hence higher *Q*-factor, for larger distance *C*_*L*_. This is reasonable as increasing *C*_*L*_ would reduce the effect of the mutual coupling between the adjacent hexagonal CSRRs.

For reliable blood glucose sensing, it is vital to maximize the resonance strength and to perfectly confine the resonating electromagnetic fields within the permittivity sensing region of the sensor. Therefore, the design and geometrical parameters of the planar transmission line as well as the engraved CSRR cells have been optimized to obtain a steep transmission resonance around *f*_0_ = 3.0 GHz for the unloaded sensor after studying the primary influence of each parameter using a Finite Element Method (FEM)-based numerical simulator as shown in Fig. 2. To elaborate, the resonance characteristics of the proposed sensor are basically determined by the substrate dielectric specifications and geometries (i.e. width and height). In addition, the design parameters of each hexagonal unit-cell play a crucial role in defining the complete resonance profile for the sensor. These parameters include the diagonal length *a* and *b*, coupling-gap *t*, side-width *s*, split-gap *g*, and coupling-controllers (*C*_*L*_ and *C*_*W*_) of the integrated honey-cell. In contrast, before engraving the hexagonal CSRRs, these resonance characteristics are solely determined by only the geometrical width and length of the dielectric patch.

### Glucose detection mechanism

A simple lumped element model is used to describe the physical behaviour of the integrated CSRR when used for glucose sensing^49,50^. This electrical model consists of three parts as shown in Fig. 2a: first, the signal line (MTL) (of 1.5 mm width) used for exciting the resonator is modelled by an inductance *L*_*c*_ to represent the magnetic coupling resulting from the MTL when crossing the CSRR structure. The second part is the dielectric substrate (intermediate junction of length 66 mm, width 20 mm, and thickness 0.8 mm) used to electrically couple the MTL with the resonators in the ground plane. This part can be approximately modelled by a shunt capacitance *C*_*c*_ in parallel with a resistance *R*_*c*_ that represents both the dielectric losses of the substrate and the conductive losses of the metal strip. The last part is the honey-cell resonator (of four cells) that is modelled by an *RLC* parallel resonant circuit where the winding of the dielectric rings (of width *s*) in each hexagonal cell acts as inductance *L*_*R*_, the metallic gaps *g* and spacings *t* between rings create a parallel capacitance *C*_*R*_*,* the conductive and dielectric losses are modelled by a resistance *R*_*R*_ that takes into account the very small inductive magnetic losses associated with *L*_*R*_. When the SUT (i.e. glucose sample) is placed on top of the CSRR surface, the last resonator part in the model is modified by adding a parallel *RC* circuit that models the dielectric characteristics of the loaded glucose sample. In specific, *C*_*M*_ is directly related to the relative permittivity of the sample, while *R*_*M*_ is mainly dependent on its loss properties. This configuration of the lumped elements stores an oscillating electric and magnetic energy in the inductance and capacitance that are arisen by the induced charges and currents inside the patterned dielectric loops or slots upon exciting the CSRRs. When both energies are in balanced state, the microwave sensor resonates at a specific frequency as given in Eq. (1), directly observed on the minimum frequency response of the transmission coefficient S_21_ with a loaded *Q*-factor given by Eq. (2)

1 $${f}_{R}({{\epsilon }_{r}}^{^{\prime}})=\frac{1}{2\pi \sqrt{{L}_{R}{(C}_{e}({{\epsilon }_{r}}^{^{\prime}})+{C}_{c})}},$$

2 $${Q}_{R}({{\epsilon }_{r}}^{^{\prime}},tan\delta )=\frac{{f}_{R}}{{\Delta f}_{3dB}}={2\pi {f}_{R}R}_{P}\left(tan\delta \right)\left({C}_{e}({{\epsilon }_{r}}^{^{\prime}})+{C}_{c}\right),$$

where ${\Delta f}_{3dB}$ denotes the 3 dB bandwidth of the resonance peak, *C*_*e*_ ~ *C*_*R*_//*C*_*M*_ denotes the effective capacitance of the integrated CSRRs when loaded by the glucose samples of various concentrations^49^. *R*_*p*_ is the equivalent resistance of paralleling the coupling-part *R*_*c*_ and CSRR-part *R*_*e*_ that represents the dielectric losses of the CSRR and its loaded glucose sample. In this sense, while the magnetic field is confined between the slit traces, the stored electric energy widely spread and concentrated in the CSRRs region interacts with the glucose samples placed in vicinity of the CSRRs. Consequently, subtle changes in the dielectric permittivity of the loaded glucose samples would disturb the electric field distribution, and thereby reflected in *f*_*R*_ through the perturbation of the CSRR effective capacitance *C*_*e*_. This shift in the resonance frequency is therefore a measure for determining the sample glucose concentration for a constant volume. Similarly, *R*_*e*_ is mainly dependent on the loss property of the glucose sample and therefore the contrast in *tanδ* is reflected as amplitude variations in the resonance profile. The resulting changes in resonance attributes are considered as a coded signature of the glucose sample dielectric properties, that could be correlated effectively to the corresponding glucose level through careful analysis of the modified resonance behaviour.

### Numerical analysis

**Figure 3:**
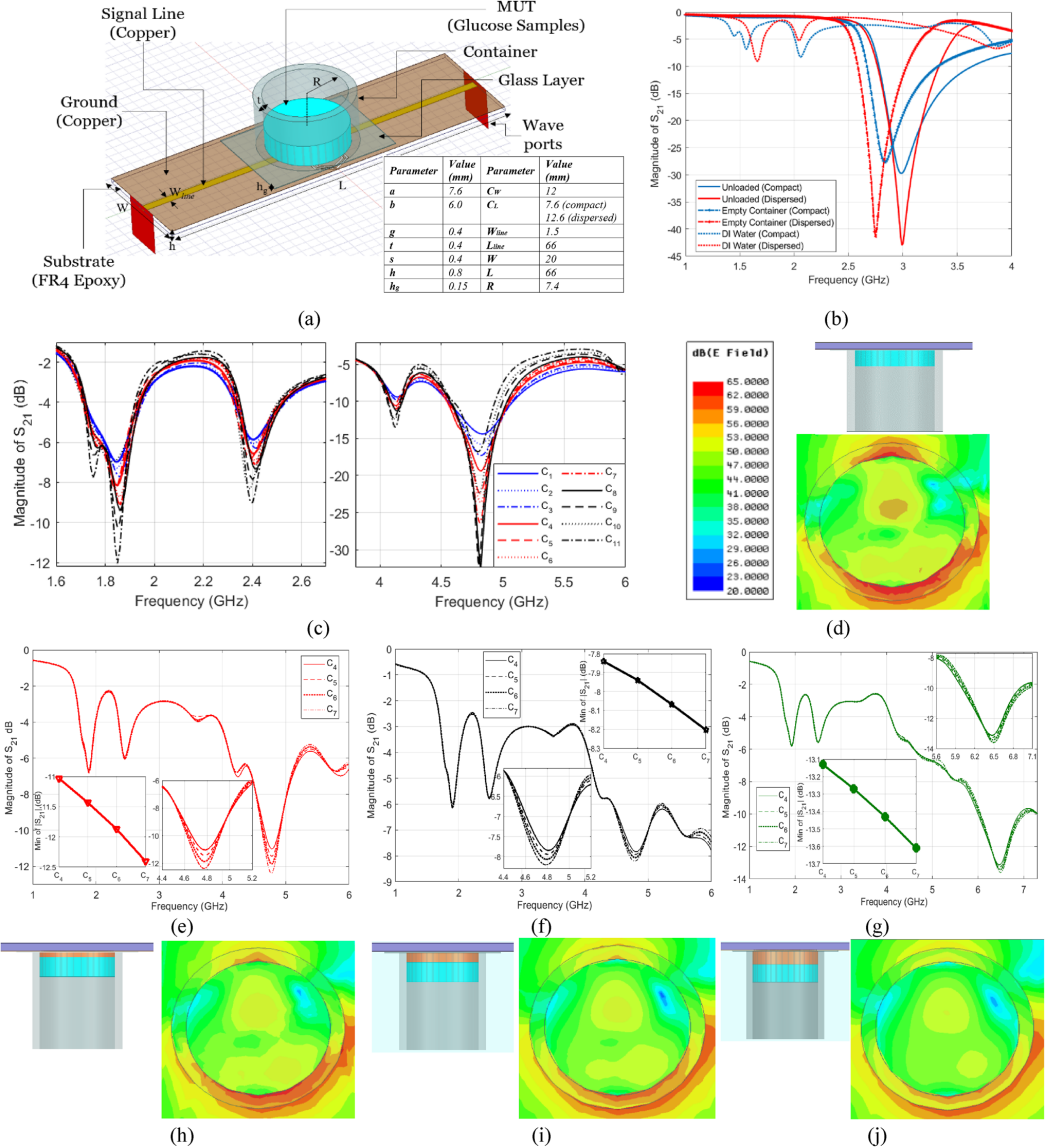
(**a**) Geometrical model and parameters of the proposed honey-cell CSRR integrated with the glass container used for loading the glucose samples, (**b**) simulated transmission response (S_21_) of the sensor topologies (compact and dispersed) for three different states: unloaded, loaded with empty container, and filled with DI-water. Simulated transmission response of different glucose samples loaded on top of the compact sensor with (**c**) no skin, (**e**)–(**g**) skin thickness of 0.5, 1.0, and 1.5 mm, respectively. Cross section of the electric field distribution in the midst of the glucose tissue in the respective cases (**d**) no skin, (**h**)*–*(**j**) skin thickness of 0.5, 1.0, and 1.5 mm, respectively.

Both topologies, compact and dispersed, were numerically analyzed using an FEM-simulator to test the functionality and quantify the resonant frequencies for three different cases: when unloaded, loaded with an empty container, and loaded with pure distilled-water (DI). A cylindrical glass container was designed to load the glucose samples onto the CSRRs surface (this is replaced later by a suitable fixture to place the fingertip) as shown in Fig. 3a. The container has 8*.*9 mm outer diameter, 7*.*4 mm inner diameter, 1*.*5 mm wall thickness, and 10 mm height. In addition, a thin glass layer of *h*_*g*_ = 0*.*15 mm was introduced between the container and CSRRs in order to evade short-circuiting the dielectric slits of the CSRR cells when loaded with the glucose specimens of moist nature. The intrinsic transmission resonant frequency prior-loading is 3.0 GHz for both topologies as shown in Fig. 3b. Loading the sensors with the empty cylindrical container will introduce a few MHz shifts in resonance as can be clearly observed in Fig. 3b, where *f*_*R*_ for the compact and dispersed are tuned to 2.83 GHz and 2.75 GHz, respectively. The empty container response will be used as a reference state when the sensors are used for sensing the glucose concentrations of interest. Filling the container with the DI-water of volume 600 µL will disturb the electric field generated in the sensitive area of the dielectric substrate, thereby resulting into further shift in *f*_*R*_ due to the change in effective permittivity of the media surrounding the resonators. A frequency shift of about 770 MHz (compact) and 710 MHz (dispersed) was noticed when the DI-water was simulated inside the container. Moreover, a damping effect was observed on these resonances with a significant drop in the resonance peaks due the lossy nature of the DI-water sample. Furthermore, an additional resonance is established around 1.56 GHz (compact) and 1.66 GHz (dispersed) as a result of this new configuration of the sensor structure that accommodates the DI-water sample.

Next, glucose aqueous samples of concentrations C_1_–C_11_ in the range 40–500 mg/dL were simulated on top of the sensing region inside the glass container. This range of concentrations covers the wide diabetes spectrum including hypoglycemia (< 70 mg/dL) and hyperglycemia (> 140 mg/dL). The single-pole (first order) Debye model given by Eq. (3) was used to construct the numerical models for the dispersive dielectric properties of the glucose-water tissues at different concentrations. It is noteworthy to mention, this model was developed in^48^ based on the spectroscopy measurements of watery solutions of 50, 250, 1,000, and 2,000 mg/dL as collected using a commercial coaxial probe kit connected to VNA. This is the most rational model to use for approximating the blood-glucose behaviour since no other mathematical model is yet developed for the dielectric properties of actual blood-glucose solutions.

3 $${\epsilon }_{r}\left(w,\xi \right)={\epsilon }_{\infty }\left(\xi \right)+\left(\frac{{\epsilon }_{stat}\left(\xi \right) - {\epsilon }_{\infty }\left(\xi \right)}{1 + j\omega \tau (\xi )}\right)+\frac{{\sigma }_{s}}{j\omega {\epsilon }_{o}},$$

where ${\epsilon }_{r}\left(w,\xi \right)$ is the complex permittivity of the aquatic solution of glucose concentration $\xi$ (in mg/dL) at angular frequency $w$, while ${\epsilon }_{stat}$, ${\epsilon }_{\infty }$, and $\tau$ are the concentration-dependent Debye coefficients^46^, ${\sigma }_{s}$ is the static conductivity, and ${\epsilon }_{o}$ is the permittivity of free-space.

Figure 3c shows the simulated sensor’s transmission responses for the different glucose samples C_1_–C_11_ loaded at volume 600 µL each (corresponds to 2 mm thickness inside the container). Four resonance frequencies were induced in the frequency range 1.5–5.0 GHz. All these resonances show variations in both frequency and amplitude for glucose level changes. However, the fourth harmonic resonance around 4.8 GHz is more responsive to the dielectric contrast of the glucose samples. This is reflected not only in the slight shift in resonance frequency, but also more distinctly in the significant change in resonance amplitude, implying that the loss property of the dissolved glucose contributes more to the system frequency response. The simulated response also demonstrates the sensor capability for glucose detection over the whole diabetes range including the hypoglycemia concentrations 40–60 mg/dL (C_1_–C_3_, shown in blue), normal condition concentrations 70–140 mg/dL (C_4_–C_7_, shown in red) and hyperglycemia concentrations 200–500 mg/dL (C_8_–C_11_, shown in black).

To mimic the realistic scenario of placing a fingertip onto the sensing region, we performed another simulation where the glucose samples C_4_–C_7_ of thickness 2 mm each were placed on top of a skin layer (${{\epsilon }_{r}}^{^{\prime}}$ = 38.1 and *tanδ* = 0.28)^51,52^ of varying thicknesses 0.5, 1.0, and 1.5 mm, and the corresponding transmission responses were analyzed over the frequency range 1–6 GHz as depicted in Fig. 3d–f, respectively. In fact, skin thickness is generally related to several factors such as body site, gender, skin type, age, pigmentation, etc., however, the simulated thicknesses are within the practical range of human fingertips^53^. It is observed that three distinct resonances were induced in these scattering responses around *f* = 1*.*9–1*.*95 GHz, *f* = 2*.*44–2*.*51 GHz and *f* = 4*.*77–6*.*5 GHz. The third resonance in each response conveys more information about the glucose concentrations of the simulated samples buried under the skin. This is demonstrated very clear in the larger amplitude variations compared to those existed at lower resonant frequencies as plotted in the enclosed graphs. However, skin layers of larger thickness would damp the corresponding resonance strength, and therefore weaken the coupled electric field that interacts with the glucose sample as shown in Fig. 3g–j that compare the electric field intensity coupled to the glucose tissue in the respective cases. Consequently, a lower sensitivity is reflected in the resonance readings with reduced amplitude resolution for sensing the glucose concentrations. For instance, by calculating the amplitude variation per unit change in ${\epsilon }_{r}^{^{\prime}}$ and $tan\delta$ for the two cases of 0.5- and 1.0-mm skin thickness, we got ∆|S_21_|/∆|${\epsilon }_{r}^{^{\prime}}$|of about 4 and 1, respectively. While that for ∆|S_21_|/∆|$tan\delta$|is around 40 and 10, respectively.

### In-vitro experiments

Various measurements have been conducted to verify the performance of the proposed glucose sensor using two different setups incorporating either (i) VNA or (ii) 2.45 GHz radar board. For the sake of simplicity, aquatic glucose solutions were used in these experiments to imitate the blood behaviour at different glucose concentrations (70–120 mg/dL) of clinically relevant to Type-2 diabetes. This would also help to secure the reproducibility of measured scattering data. Many other studies have also adopted the glucose aqueous solutions for preliminary experiments on non-invasive glucose detection using RF sensors^54–56^. This approximation is valid since water contributes about 50% of the entire human blood that contains other vital components at varying proportions (e.g. Na, Ca, Mg, K, Cl, etc.). These minerals are present at lower concentrations compared to the dominant glucose whereby the blood dielectric properties are dominantly affected^46,57–59^. In particular, compared to the broad range of glucose concentrations in blood; other minerals only vary in limited ranges, Na: 310–333 mg/dL, Cl: 337–372 mg/dL, Mg: 1.8–3.4 mg/dL, Ca: 8.5–10.5 mg/dL, K: 13.6–21.4 mg/dL)^59^, and other components in blood like the lactic acids, have also very small concentrations (normal range 4.5–19.8 mg/dL) when compared to the dominant glucose. Moreover, Na and Cl that exist in larger order of magnitudes compared to other minerals would mostly influence the conductivity of the blood and therefore their effect is negligible on the frequency shift in comparison with the effect of glucose variation. Since the frequency shift is considered as the main sensing parameter of the sensor, it is reasonable to assume that the small mineral concentration variations do not interfere with results from glucose related frequency shift.

#### Measurements using the VNA

**Figure 4:**
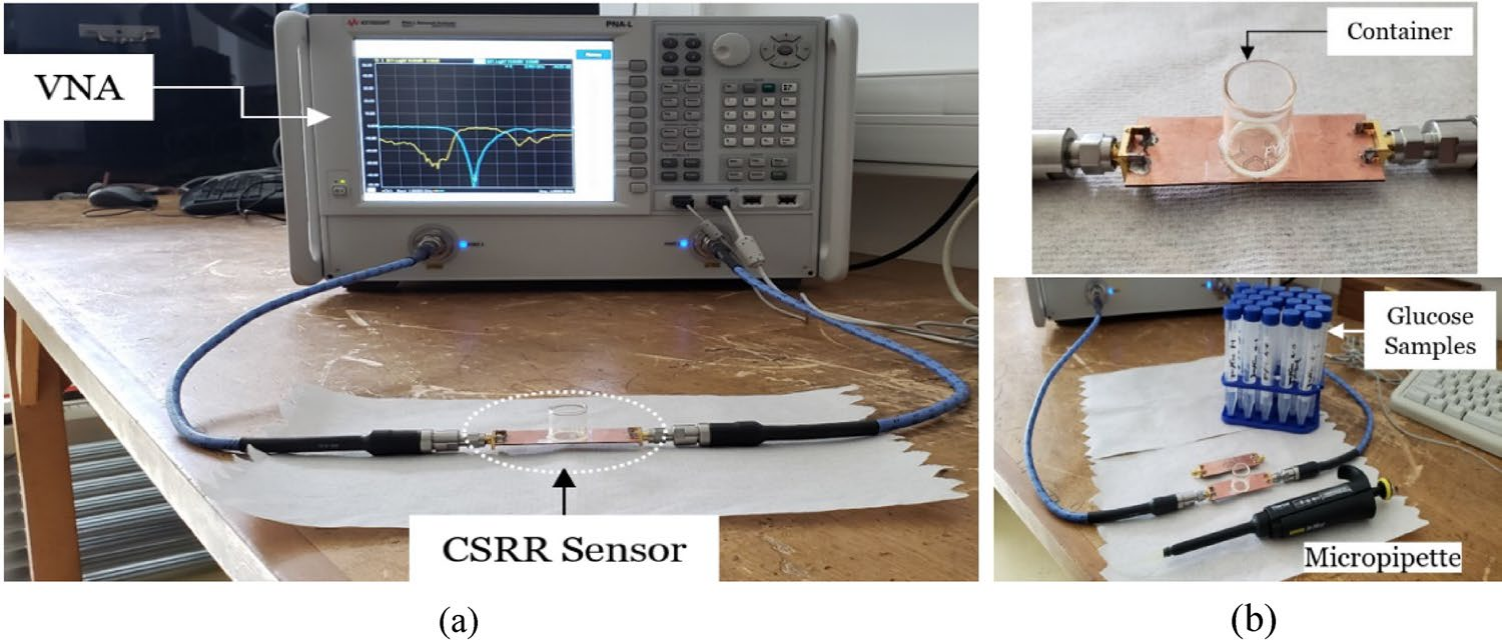
Glucose sensing experiments (**a**) VNA experimental setup, (**b**) sensor loaded with an empty container, the micropipette and glucose aqueous solutions used in the experiments.

**Figure 5:**
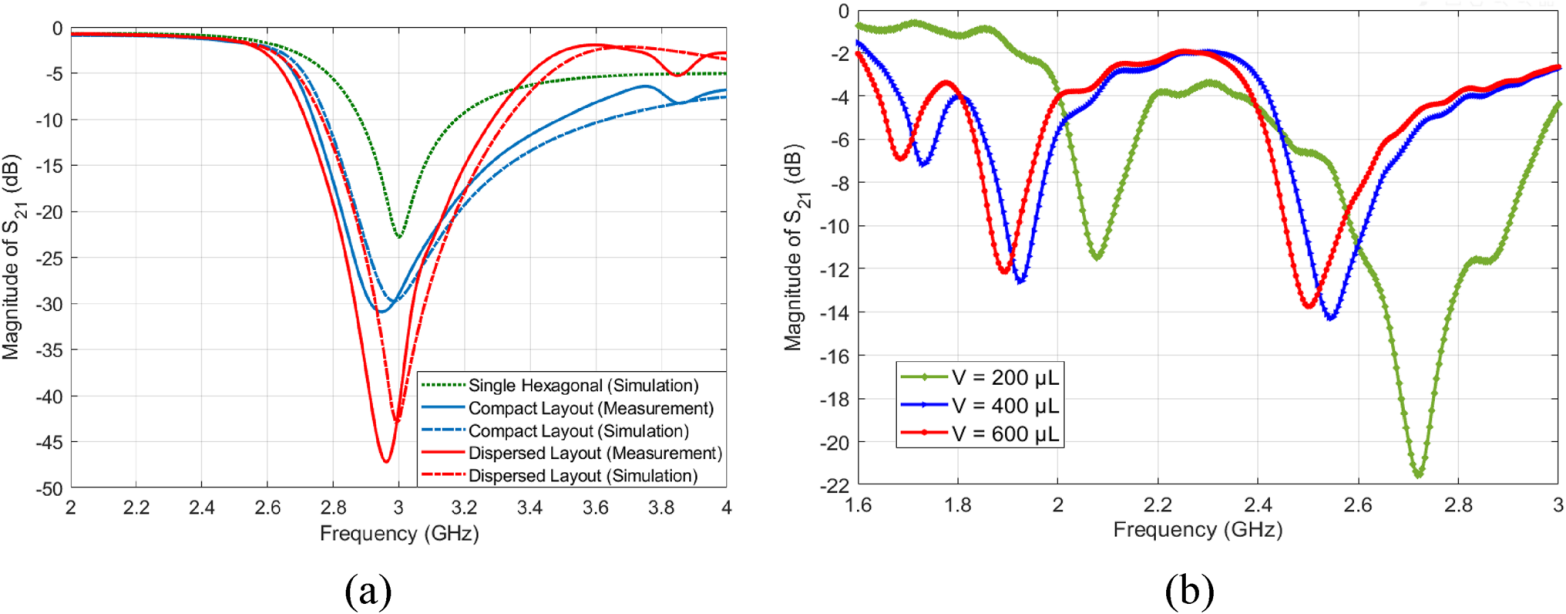
(**a**) Measurements and simulations of the transmission coefficients of the fabricated sensors before loading glucose samples (resonance around 3.0 GHz), (**b**) measured transmission coefficient S_21_ when the compact sensor is experimented for the 70 mg/dL glucose sample at different volumes 200, 400, and 600 µL to compare the corresponding sensor behaviors. Increasing the sample volume, and implicitly the thickness, would tune the S_21_ resonant frequencies towards lower frequencies.

First, the fabricated prototypes (compact and dispersed) were experimentally tested using the VNA setup shown in Fig. 4a to record the intrinsic transmission responses before loading any glucose sample. Figure 5a presents the measured transmission coefficients as a function of frequency for the unloaded compact and dispersed sensors while comparing against the conventional single-hexagonal CSRR (shown in dotted green). Under unloading conditions, the three CSRR structures experience transmission resonances around *f*_*R*_ = 3*.*0 GHz with resonance minima of about − 22.85 dB, − 29.57 dB, and − 43 dB, for the single-hexagonal, compact, and dispersed, respectively, as depicted in Fig. 5a. Both compact and dispersed sensor have steeper resonance depth that would provide a better sensitivity for characterizing lossy glucose samples of high *tanδ* (i.e. detecting the tiny variations in the loss tangent for various glucose concentrations). These measurement results show an excellent agreement with the predicted numerical simulations. Minor differences are due to typical tolerances in the fabrication process.

**Figure 6:**
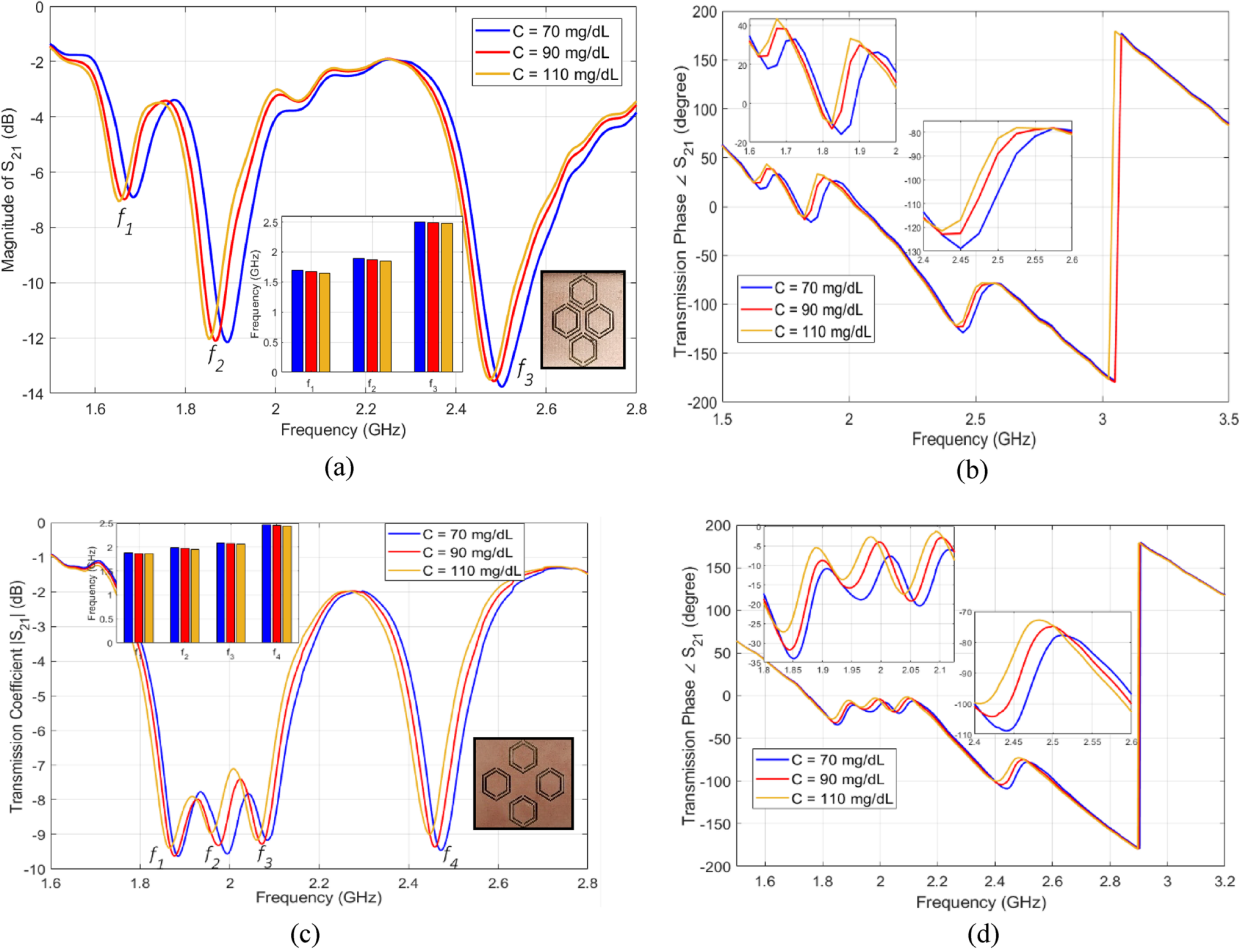
Measured transmission response S_21_ as function of frequency for tested glucose samples of various concentrations. The transmission coefficient and phase for the (**a**), (**b**) compact and (**c**), (**d**) dispersed sensors, respectively.

**Figure 7:**
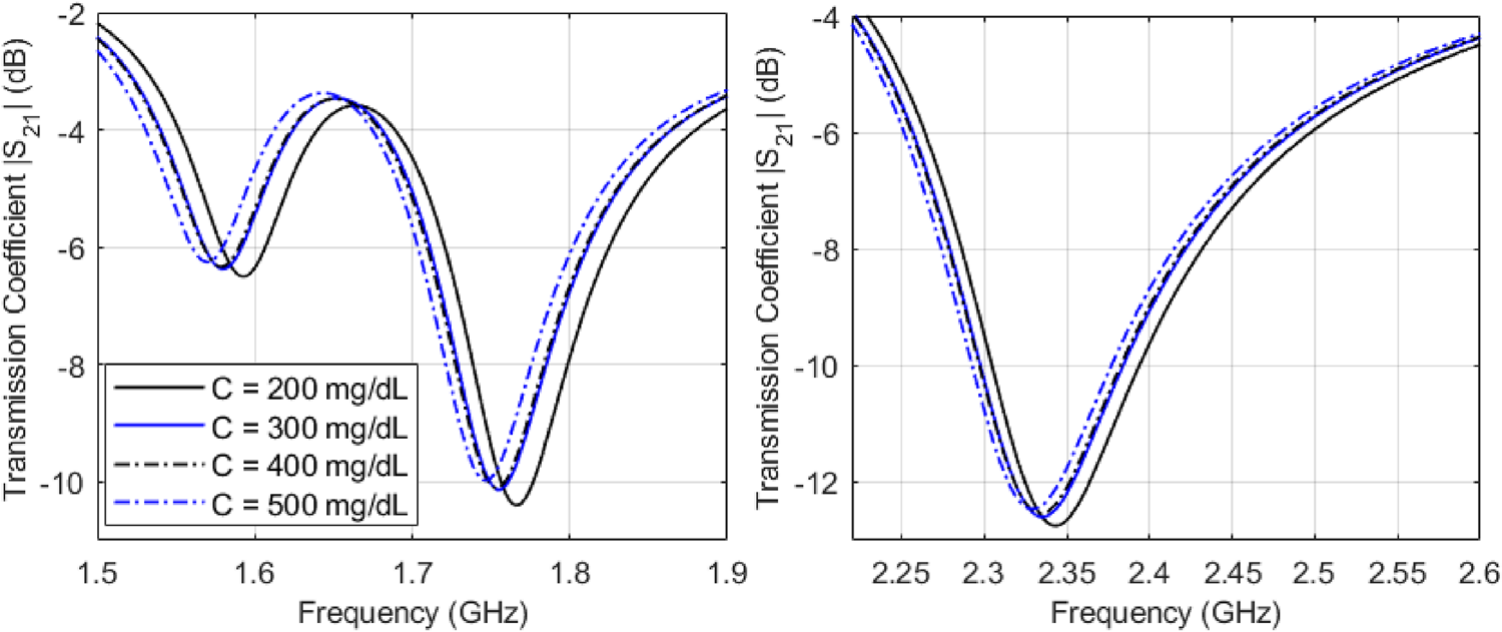
Measured transmission response |S_21_| as a function of frequency for tested glucose samples of higher concentrations of clinically relevant to the hyperglycemia condition in diabetes.

In order to test the developed sensors for measuring glucose samples at the levels of interest, we fabricated a cylindrical glass container to hold the samples on top of the CSRRs surface. The empty cylindrical container was placed on the honey-cell CSRR structure, as shown in Fig. 4b. This will introduce a few MHz shifts from the reference resonance in S_21_ of the unloaded state. In each measurement trial, the micropipette device was used to measure a precise volume of *V* = 600 µL from each concentration, load it inside the container and the change in the transmission resonance frequency response was recorded. This volume was adopted for testing various glucose samples to minimize the measurement errors due to uncertainty in the sample volume. In this regard, the sensor response was studied at different volumes of the tested sample as shown in Fig. 5b. It is observed that the sensor shift in resonance frequency would converge to a lower limit as the sample volume amount to 600–700 µL which enables a homogeneous distribution of the sample inside the container whereby the sensing region is completely covered. To test the sensor response for another concentration, a clean tissue paper was used to completely remove the previously tested glucose sample with attention to retrieve the exact reference resonance at S_21_ prior-loading the sample for a fair comparison. Figure 6a,b show the transmission response (magnitude and phase) of the compact sensor when the concentration of glucose is changing in 70, 90, and 110 mg/dL. Figure 6c,d capture the same responses when the dispersed sensor was used for testing. In both sensors’ responses, it was observed that the resonant frequencies at which the transmission is minimized are shifted towards lower frequencies as the glucose concentration in the sample increases. Additionally, trackable amplitude changes are exhibited in some resonances due to the tiny variations in the loss tangent of the tested glucose sample. Minor variations were also noticed in the transmission phase responses as shown in Fig. 6b,d. Similar observations on the resonance frequency and amplitude were recorded when the compact sensor was tested for sensing higher glucose concentrations in the range 200–500 mg/dL that represent the hyperglycemia condition as shown in Fig. 7.

#### Sensitivity analysis

**Figure 8:**
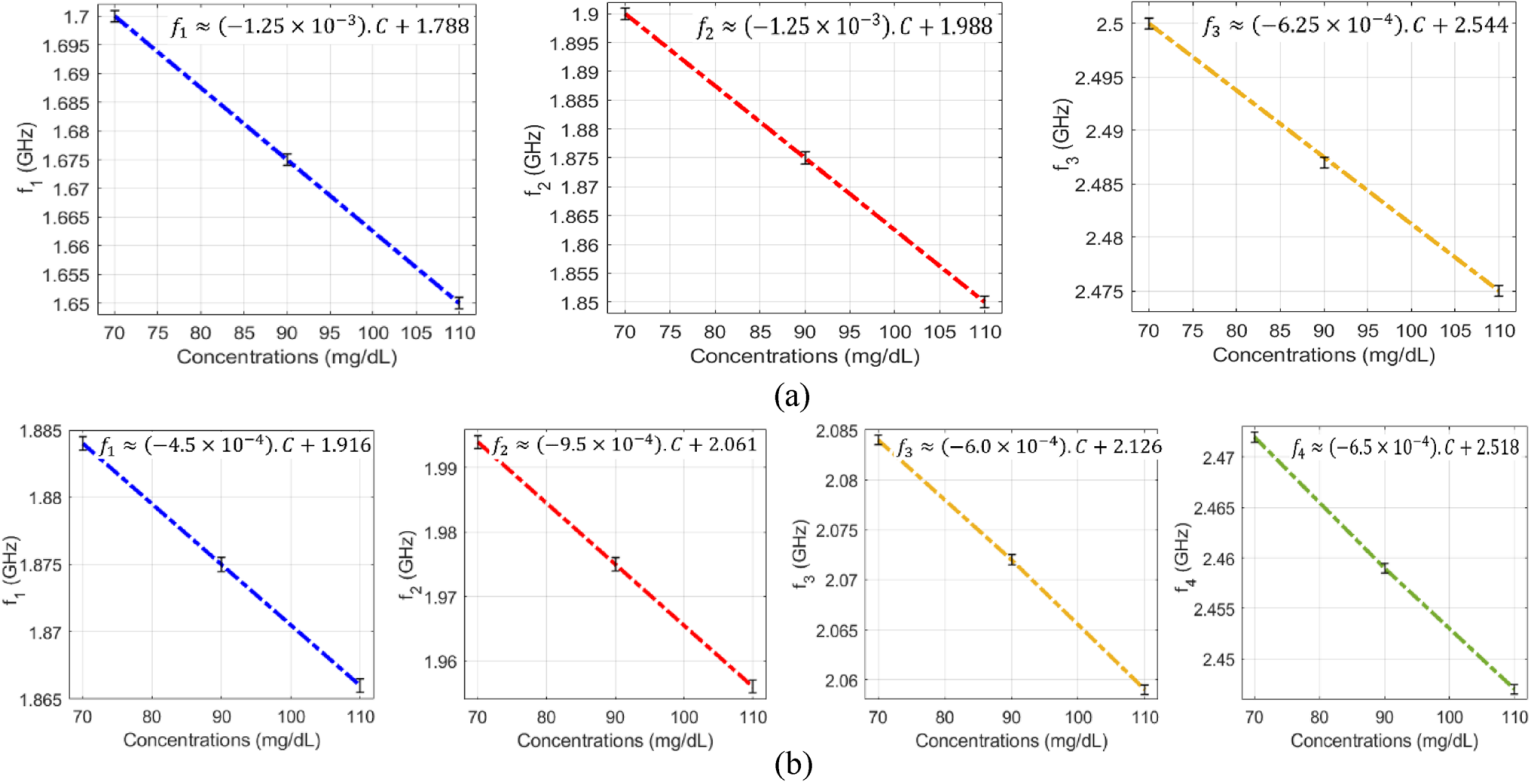
Linear correlation models for the resultant resonant frequencies of (**a**) compact and (**b**) dispersed sensors at different glucose concentrations. (**a**) For the compact sensor, three resonances, *f*_1_*, f*_2_*,* and *f*_3_, are established in the frequency range 1.5–2.8 GHz. Sensing the glucose samples on (**b**) The dispersed sensor exhibits four distinct resonances *f*_1_, *f*_2_, *f*_3_, and *f*_4_ in the frequency range 1.6–2.8 GHz. A reduction in the resonant frequencies is observed with growing glucose concentrations.

The VNA measurements of the scattering-parameters of the glucose-loaded CSRR sensors show that both reflection and transmission responses (magnitudes and phases) are varying according to the glucose level changes in the loaded sample. Particularly, considerable shifts in the resonance frequencies of the S_11_ and S_21_ are observed for varying between 70, 90, and 110 mg/dL. The data sheet of the VNA instrument used in this experiment poses for higher uncertainties in the readings of the reflection coefficient (magnitude and phase) compared to the transmission coefficient that is more accurate and stable. For this reason, we only considered the frequency shifts in the transmission coefficient S_21_ to assess and compare the sensitivity range of the proposed sensors. As seen from Fig. 8a,b, both sensors exhibit impressive frequency resolutions that would be beneficial to distinguish various glucose concentrations of relatively small contrast in dielectric properties. The resonance frequencies resultant from loading a specific glucose concentration can be estimated using the linear regression models derived for each sensor, compact and dispersed, as shown in Fig. 8a,b, respectively. Conversely, the inverse models could be used to estimate the unknown glucose level of a tested sample. In these plots, it is observed that the respective resonant frequencies decrease with increased levels of glucose concentrations. However, the frequency resolutions for glucose level changes at the respective resonances are not perfectly identical. The sensitivity of the compact sensor is estimated as − 1.25 × 10^–3^ GHz/(mg/dL) representing the gradient of *f*_1_ and *f*_2_ linear models. However, the best sensitivity slope for the dispersed topology is recorded as − 9.5 × 10^–4^ GHz/(mg/dL) at *f*_2_ which is a bit lower than the compact counterpart.

**Figure 9:**
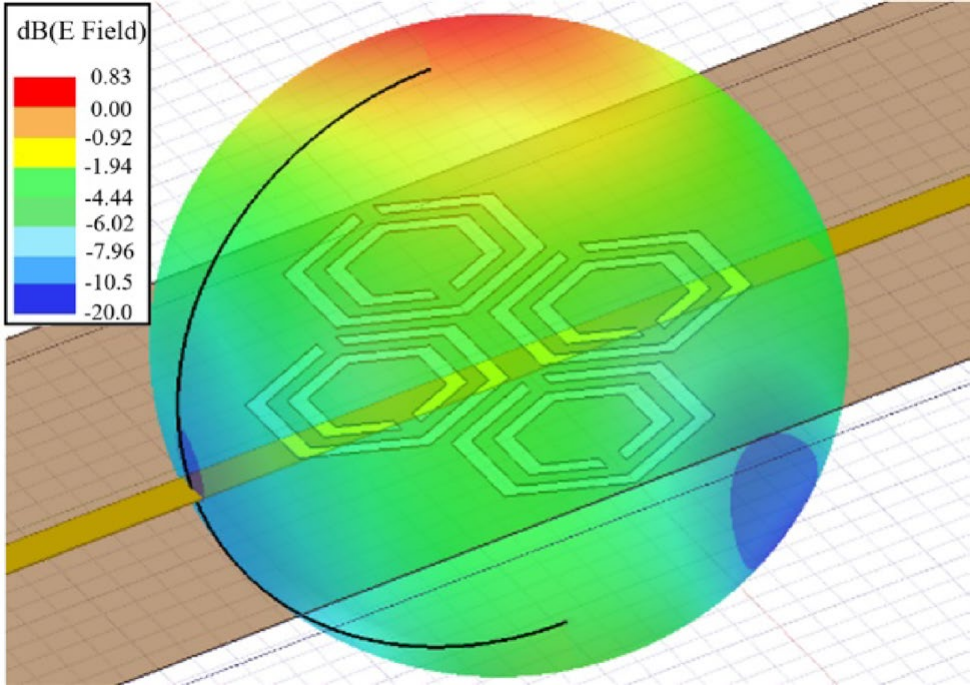
Far-field radiation of the CSRR sensor.

The VNA microwave system was strictly calibrated to limit the transmission loss at 0.02 dB over the operating frequency range. This is important to acquire reproducible measurements that are more accurate. It is noteworthy to mention that, all the glucose measurements were repeated three times and the average is reported for precision, repeatability, and reproducibility verifications. This averaging would help to eliminate random noise introduced by the source power (i.e. VNA) or any other uncorrelated noise and improve the SNR accordingly. In each repeatability trial, careful attention was paid towards retrieving the exact initial resonance response of the empty container-loaded sensor after removing the samples using a tissue paper. The robustness of the sensor could be seen from the stable and repeatable frequency responses that vary in a small range of ± 0.5 MHz as indicated by the error bars enclosed in these visuals. Given that the sensor frequency reading for 5 mg/dL is about 4.7 MHz (average sensitivity of 0.94 MHz/(mg/dL)), the glucose concentrations as small as 1 mg/dL could be identified using the proposed sensing platform with a reasonable accuracy. Since the sensor scattering response is quite dependent on the sample EM properties which are temperature dependent, therefore, we stored all the prepared glucose samples in the same room of temperature regulated at 25 ± 1 °C. Additionally, the heat and temperature were controlled and monitored at 25 ± 1 °C during the glucose experiments to minimize the instrumental and environmental impact on the collected measurements. However, small fluctuations in temperature will not bring a significant effect on the resonance measurements of the CSRR sensors (± 1 MHz shift in *f*_*R*_). This is explained by the fact that, the far field radiation of our small resonators of sub wavelengths could be effectively suppressed as depicted in Fig. 9 where the total electric field is quantified in the far field region of the sensors with maximum magnitude of about 1 V/m. Previously, it is shown that most of the electromagnetic energy is concentrated in the honey cell area in the near-field region. Therefore, any unwanted environmental reflections will not have notable effect on the sensor measurements.

Lastly, in what follows the sensitivity performance of the proposed honey-cell sensors is compared to other microwave sensors in the recent literature. To this end, a comparative statement is presented in Table 2 where state-of-the-art glucose sensors are sorted in the order of increasing sensitivity with their respective parameters. Herein, the sensitivity is defined as the frequency shift/variation ∆*f*_*R*_ with respect to 1 mg/dL of glucose concentration change in SUT for a certain volume and specific test setup. Following this measure, the sensitivity achieved in earlier works based on diverse microwave sensing mechanism is much lower than the least resolution adopted by our proposed sensors. The sensitivity of the proposed sensors is also superior to other techniques based on tracing the slight variations of S_11_ and S_21_ resonant magnitudes for which a measuring tool of high-precision is needed. The limited sensitivity in the literature is due to the confined EM fields coupled to the resonator surface that limit the interaction with the tested glucose sample. Because of this phenomenon, the substrate in traditional resonators has a more important role in defining the resonance frequency rather than the SUT of glucose content. However, the improved design of the CSRR elements in the presented work, where this interaction is greatly expanded over the sensing region, allows the resonance frequency response of the sensor to be mainly defined by the SUT permittivity. To the best of our knowledge, the achieved sensitivity of this work, 0.94 MHz/[mg/dL] is far beyond the best results reported in literature regardless of shape and volume of SUT. This would render the sensor response less susceptible to environmental and instrumental noises than its conventional counterparts. Therefore, it can be emphasized that the proposed sensor can in principle be used to detect normal blood sugar range quite conveniently as well as those for hypoglycemia and hyperglycemia. It could also be used as a warning tool to diabetic patients when attempting to consume energy drinks and fruit juices which have inherently high glucose concentrations.

**Table 2:** Comparison of different microwave glucose sensors.

Ref	Sensing technique	Operating frequency (GHz)	Test solution	Sample volume (µL)	Concentration (mg/dL)	Sensing parameter	S (per MHz/[mg/dL])
^60^	Microstrip based series resonator	6.5	Aqueous solution	50	0–70,000	*f*_*R*_ (*S*_21_)	3.13E−05
^38^	Spatially separated SRR	1.4	Aqueous solution	600	504–3531	*f*_*R*_ (*S*_21_)	1.83E−04
^55^	Dielectric resonator	1.68	Aqueous solution	5	5000–30,000	*f*_*R*_ (*S*_11_)	2.00E−04
^61^	Rectangular waveguide cavity	1.9	Aqueous solution	43–170	500–2500	*f*_*R*_ (*S*_21_)	4.00E−04
^62^	Patch antenna	5.0	Saline-glucose solutions	36,000	0–250	*f*_*R*_ (*S*_11_)	1.09E−03
^63^	Open split ring resonator	6.5	Aqueous solution	–	0–40,000	*f*_*R*_ (*S*_21_)	1.88E−03
^40^	ENG unit-cell resonator	2.074	Aqueous solution	2	2000–10,000	*f*_*R*_ (*S*_21_)	1.00E−02
^64^	Single-port resonator	4.8	Aqueous solution	125	100–1000	*f*_*R*_ (*S*_11_)	1.40E−02
^65^	Distributed MEMS transmission lines (DMTL)	16	Aqueous solution	–	0–34,780	*f*_*R*_ (*S*_11_)	1.64E−02
^66^	Interdigitated capacitor (IDC) resonator-etched coplanar waveguide (CPW)	2.46	Aqueous solution	2	0–8000	*f*_*R*_ (*S*_21_)	2.00E−02
^41^	Complementary electric-LC (CELC) resonator	1.0–1.70	Aqueous solution	0.63	2000–10,000	*f*_*R*_ (*S*_21_)	2.11E−02
^67^	Split ring resonator	4.18	Aqueous solution	–	0–5000	*f*_*R*_ (*S*_21_)	2.60E−02
This work	Complementary split ring resonator driven by an ISM Radar	2.45	Aqueous solution	600	40–140	*f*_*R*_ (*S*_21_)	4.5–9.50E−01 (dispersed) 6.3–12.5E−01 (compact)

#### Machine learning processing

The sensor’s scattering responses collected from the VNA instrument are analyzed using the Principal Component Analysis (PCA) unsupervised machine learning algorithm to clearly distinguish various glucose concentrations tested by the sensor. This is a vital add-on to the integrated microwave sensor that could further enhance its detection sensitivity for the blood glucose concentrations of interest that vary in a narrow range representing the normal diabetes condition. It is shown in the previous experimental results that the sensor exhibits distinctive scattering responses in terms of the transmission coefficient (magnitude and phase) when loaded by various glucose concentrations, however these responses recorded by the VNA (i.e. |S_21_| or ∠S_21_) would exhibit small changes/shifts in *f*_*R*_ to reflect the varying glucose concentrations of the tested samples (e.g. Fig. 6a,c). For instance, the slight frequency shifts acquired by the VNA for the compact |S_21_| resonances that vary in a small resolution of 1.25 MHz/(mg/dL) as shown in Fig. 8a. These changes would be tricky to trace using exclusively common sense and hence the difficulty of precise identification of various glucose levels. The PCA as a robust classification algorithm can be used to further analyze the raw data induced by the sensor for different glucose samples. In particular, to enhance the small scattering differences between various glucose levels, we used to apply the PCA algorithm that maps the feature vectors (measured scattering data) for each glucose sample into a two-dimensional space where each glucose concentration is represented by only two indices called the principal components $\mathcal{K}$. This will help to bring a higher resolution when the sensor’ responses are correlated to different blood glucose levels. In other words, the PCA algorithm creates a mapping between 1) X ∈ R^N^: the N-dimensional input variables’ vector (also referred to as the features’ vector) which corresponds to the measured |S_21_| or ∠S_21_ obtained at the different frequencies over the operating frequency range and 2) *y* ∈ R: the outcome variable which corresponds to the estimated glucose level.
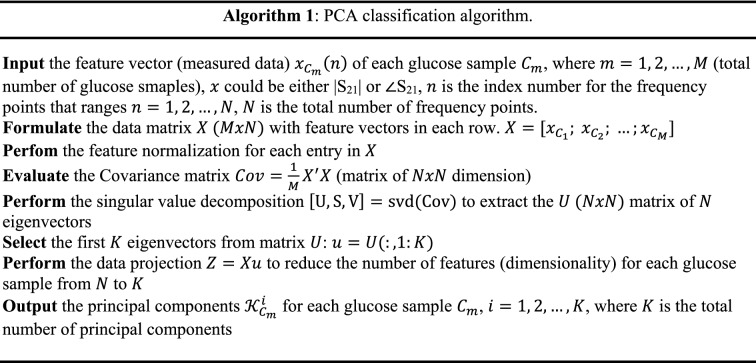


The theory of the PCA algorithm is based on the dimensionality reduction of the problem where a vector space transform is performed^68^. Herein, the PCA is exploited to extract important features from the data set of the sensor scattering responses and further to express this information as a set of orthogonal variables called principal components^69^. Generally, these principal components are derived from the eigen-decomposition of positive semi-definite matrices and the singular value decomposition of rectangular matrices^69^. In this sense, original datasets of high dimensionality could be reduced to smaller number of distinctive variables via mathematical projection without missing much information to analyze patterns, tendencies and anomalies.

**Figure 10:**
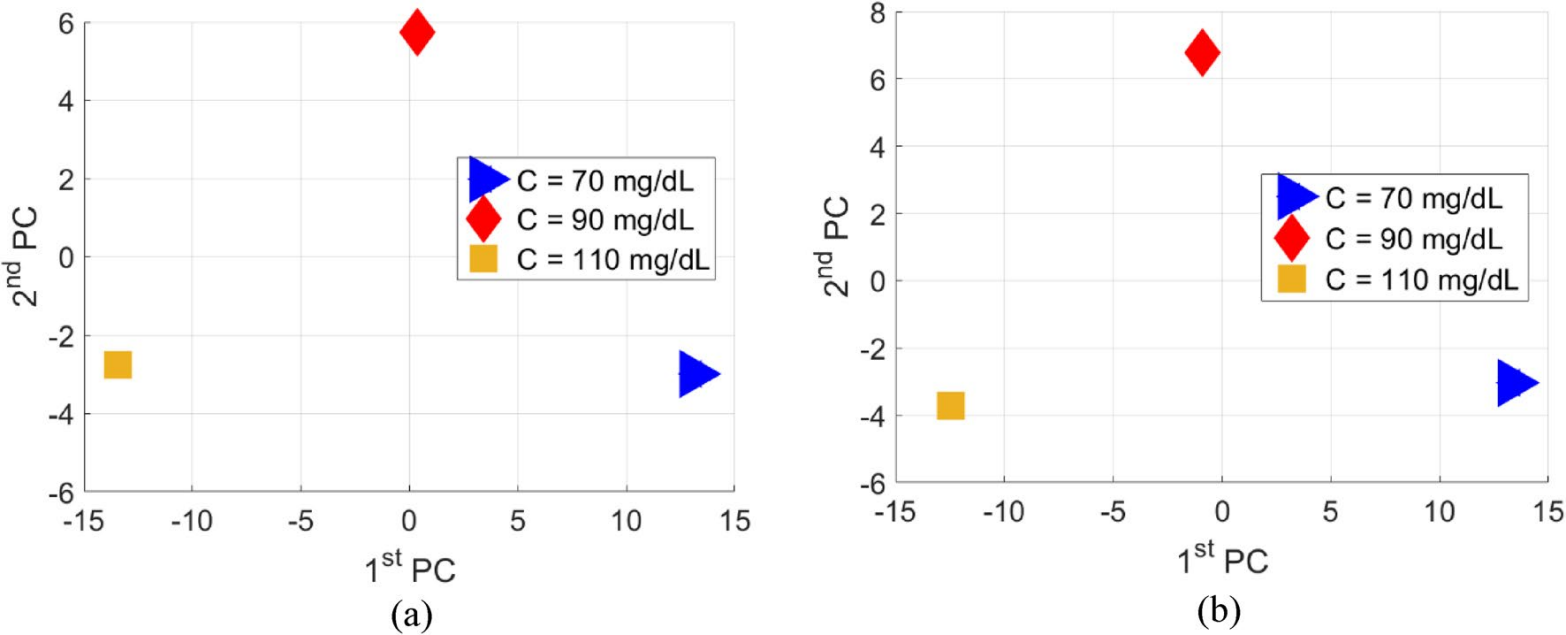
ML processing of the measured transmission responses of the compact sensor for various glucose concentrations (Fig. 6a,b), (**a**) Magnitude of S_21_ using PCA and (**b**) phase of S_21_ using PCA.

The pseudocode in Algorithm 1 describes this classification routine when applied to the VNA measurements in Fig. 6a,b, the transmission coefficient parameters (magnitude and phase) are considered the two feature vectors to be extracted from the frequency response of each tested glucose sample. Since these magnitude and phase responses were recorded at *N* = 201 frequency points spanning the range from 1.5 to 2.8 GHz, then each glucose sample is described by two feature vectors magnitude and phase with 201 length each. It is shown that samples of various concentrations have these magnitude and phase values varying at some of the respective frequency points especially at resonance frequencies. As described in Algorithm 1, the PCA algorithm could use either the magnitude (|S_21_|) or phase (∠S_21_) vectors as input features to classify various glucose concentrations. Once the algorithm is provided with measured feature vector ${x}_{{C}_{m}}$ for each glucose sample ${C}_{m}$, it processes the data using the steps in Algorithm 1 to extract the principal components ${\mathcal{K}}_{{C}_{m}}^{i}$ for all the glucose samples and cluster them accordingly based on their concentration-dependent principal components as shown in Fig. 10a,b where samples of different glucose concentrations are notably separated in the PCA space when the magnitude and phase feature vectors are used, respectively. We also observe that both feature vectors (i.e. magnitude and phase) of the sensor are effective and efficient to bring higher discrimination or spatial separation between different glucose samples. This is considered as a training stage for the developed PCA model; therefore, any new tested sample could also be clustered accordingly following the same analytical recipe.

#### Testing using the radar system

**Figure 11:**
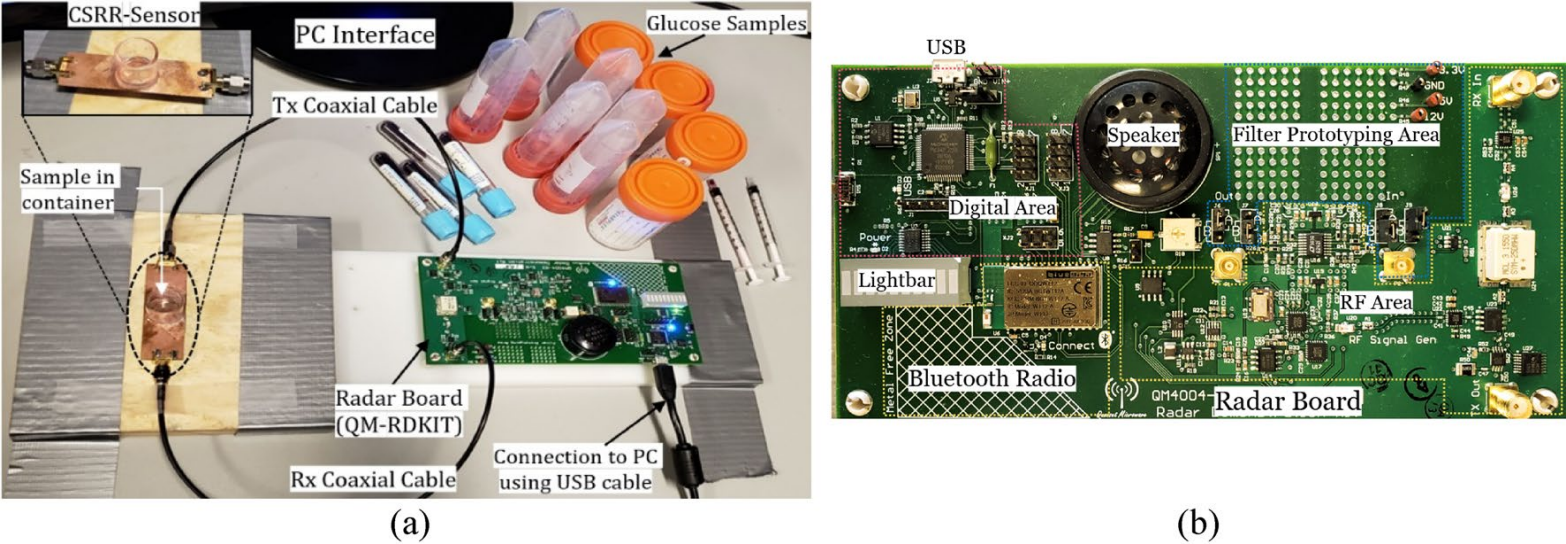
Glucose sensing experiments in (**a**) A portable setup for the honey-cell sensor connected to the low-frequency radar board, (**b**) The major sections inside the radar board: digital, Bluetooth radio, RF, filter prototyping, and lightbar for audiovisual feedback^45^.

A portable prototype of the compact sensor was developed by interconnecting a small low-cost and low-power radar module as a driving source instead of the bulky VNA instrument. In particular, the open source QM-RDKIT that supports the frequency modulated continuous wave (FMCW) functionality, was utilized to couple the CSRR sensor at the ISM frequency range (2.4–2.5 GHz)^45^. To do so, the transmitting “Tx” and receiving “Rx” ports of the radar board were connected to the feeding ports of the CSRR sensor using the SMA RF coaxial cables as shown in Fig. 11a. The major sections of the radar board are shown in Fig. 11b. It has an RF section to generate and output the transmitted signal and to downconvert the received signal to a frequency range that can be easily digitized using the onboard Analog-to-Digital converter (ADC). Specifically, the onboard Voltage Controlled Oscillator (VCO) and Phase Lock Loop (PLL) are used to generate the transmitted signal of defined frequency. The PLL serves to frequency lock the output of the VCO using the onboard reference to provide a stable and repeatable output frequency. The output of the VCO is amplified before being passed to the input port of the interconnected sensor. The corresponding signal from the sensor output port is first mixed with a sample of the transmitted signal to produce a frequency offset (beat frequency), then it is filtered to remove any unwanted signals developed from the mixing process. Afterwards, the signal is passed to the ADC for digitization, and either stored in memory or streamed over the USB/Bluetooth connection. In addition to the ADC, the digital section also contains the PIC microcontroller, USB and power interfaces. The PIC microcontroller coordinates all functions of the radar board, responds to all control commands and data requests received through the USB/Bluetooth connections.

**Figure 12:**
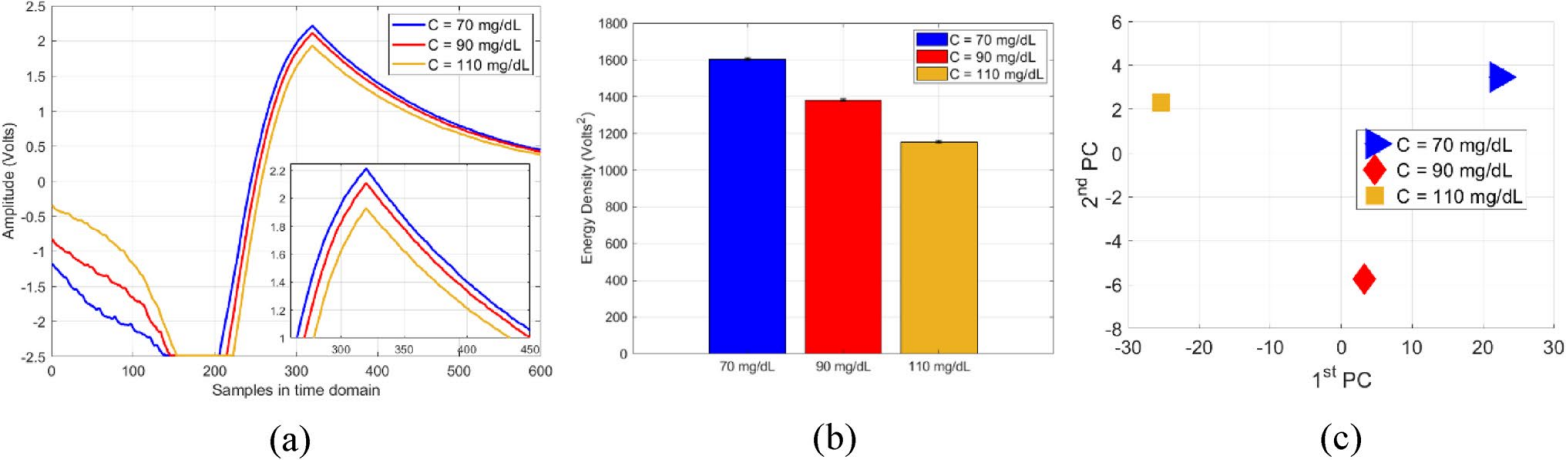
Glucose level detection using the radar setup (**a**) Raw data for tested glucose samples as collected on the receiving channel, (**b**) Comparison of energy density between different glucose levels and (c) The PCA processed results.

For glucose measurements, a micropipette was used to measure a precise volume of *V* = 600 µL from each blood sample and transfer the blood into a cylindrical container integrated on top of the sensor. Once the glucose sample is fully loaded, the RF power button was triggered from the PC interface to transmit a radar signal of one-way single sweep mode into the CSRR sensor. For repeatability verifications, each measurement trial for a glucose sample was repeated three times with a repeatability error of about ± 0.02 V. The corresponding raw data was collected accordingly and sent to the host PC over a USB connection. The data could also be sent wirelessly via a Bluetooth connection. The average of *M* = 3 repeatable voltage signals ${A}_{C}(n)=\frac{1}{M}\sum_{i=1}^{M}{x}_{C}^{i}(n)$ for each glucose sample *C* is plotted in Fig. 12a. Next, the respective average signals ${A}_{C}(n)$, *C* = 70*,* 90*,* and 110 mg/dL, were further processed to reveal the energy density corresponding to each glucose sample. Particularly, the finite energy for each average signal ${A}_{C}(n)$ of a tested glucose sample was evaluated using ${\varepsilon }_{s}=\sum_{n=1}^{N}{\left|{A}_{C}(n) \right|}^{2}$, where *N* = 600 is the total number of time samples in each average signal. The dielectric contrast of different glucose samples was distinctly captured by the sensor and demonstrated in the varying energy density 1,604, 1,382, and 1,153 Volts^2^ for the 70, 90, and 110 mg/dL, respectively, as depicted in Fig. 12b. The sensor measured data was also processed using the PCA classification algorithm that clusters the corresponding glucose samples based on their extracted principal components as depicted in Fig. 12c.

### In-vivo experiments

**Figure 13:**
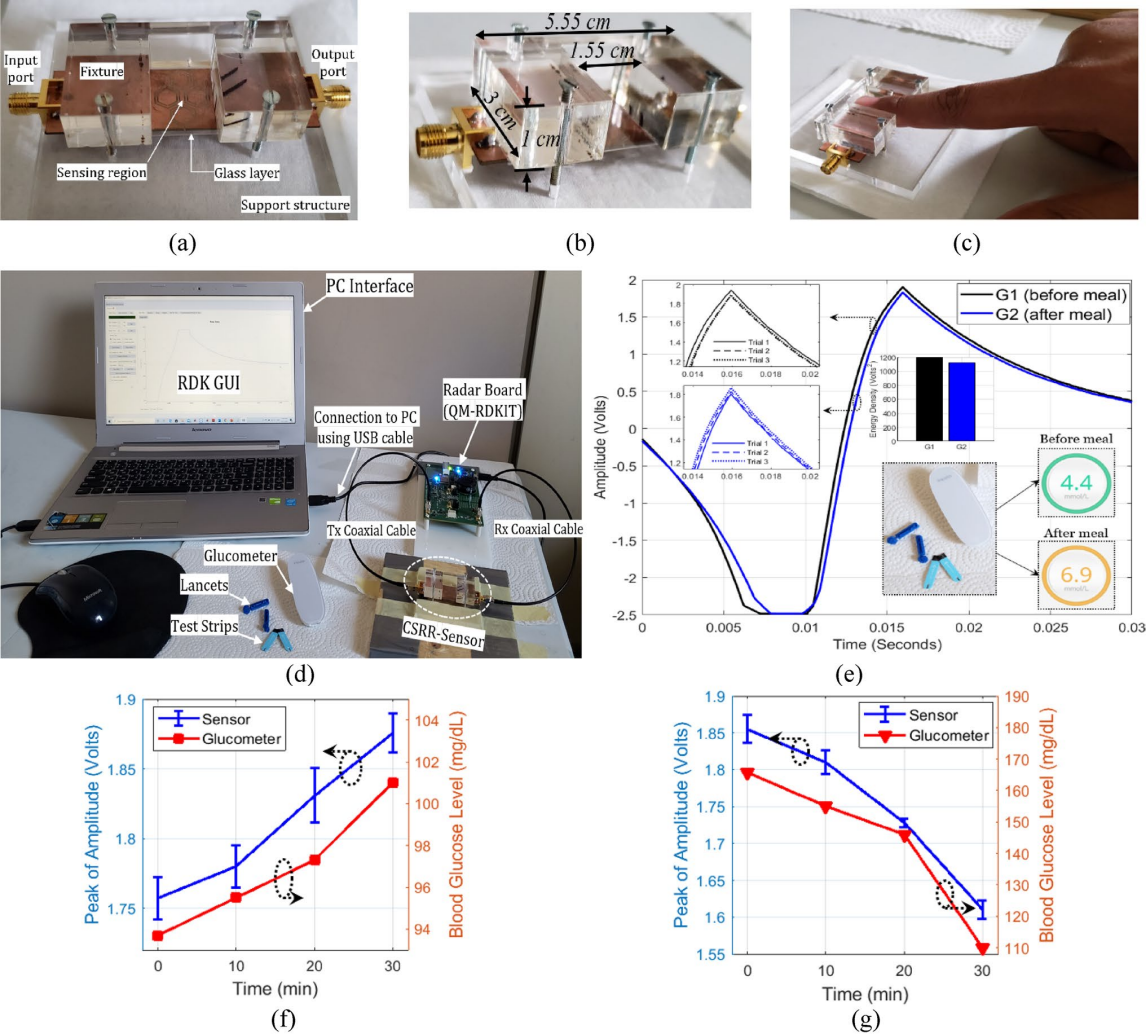
Blood glucose level tracking from an individual’s fingertip (**a**) fabricated fixture for fingertip placement, (**b**) geometrical dimensions of the fixture, (**c**) fingertip placement in the fixture, (**d**) complete setup for the in-vivo experiment, (**e**) analysis of the BGL discrete readings from the CSRR sensor and glucometer. Continuous BGL monitoring over a course of 30 min for (**f**) pre-prandial, and (**g**) post-prandial testing. The peak amplitude of the sensor response versus time (blue curve). The invasive measurements for BGL variations over time (red curve).

In the light of previous positive results, the integrated portable sensing system was further tested for a simple in-vivo experiment as a proof-of-concept for this technology when revised for intermittent or continuous blood glucose monitoring. For this purpose, the CSRR compact sensor was attached to a fixture structure suitable for finger placement as shown in Fig. 13a. The geometrical dimensions of the fixture are shown in Fig. 13b. The new prototype was integrated with the radar board (QM-RDKIT) as captured in Fig. 13d for the entire system setup. The radar system was interconnected to a laptop where the radar operation was controlled through a GUI. Preliminary tests were performed on a healthy male volunteer at age 29 before and after having the lunch meal while comparing the non-invasive measurements against a standard glucometer used as a reference for comparison. This testing recipe was guided by the fact that, in healthy non-diabetic people, the blood glucose level should measure between 72–99 mg/dL before a meal and should be less than 140 mg/dL two hours after a meal^32,33^. Therefore, a pre-prandial test was first performed for the tested subject by placing his fingertip suitably in the sensing region inside the fixture as shown in Fig. 13c. The finger should be in contact with the sensing region (firmly attached to the fixture) to perturb the electromagnetic fields and induce noticeable changes in the sensor transmission response. The sensing process from the fingertip would take a short time of about one-minute max during which no changes in the temperature status of the subject finger is expected. The corresponding raw data in response of a one-way single sweep transmission was collected from the radar receiving channel using the featured GUI. The same test was repeated three times for repeatability verification and the average of the three readings (with ± 0.03 V error max) is plotted in Fig. 13e (black curve). Afterwards, the individual’s BGL was measured using the commercial invasive glucometer to get the actual pre-prandial BGL of about 4.4 mmol/L (≈ 80 mg/dL). Similarly, a second test was performed for the tested subject two hours after having a lunch meal for normal diet. The test on the non-invasive sensor was repeated for three consecutive times and the average voltage signal is plotted in Fig. 13e (blue curve). The post-prandial BGL was measured at 6.9 mmol/L (≈ 124 mg/dL) on the glucometer. Following these measurements, the transmission results of the CSRR sensor were observed to be reliably consistent and aligned with the glucometer readings for the individual BGL variations before and after the meal. Particularly, the sensor’ transmitted signal exhibits a change in amplitude and a shift in time domain in response to the varying BGL of the tested subject. The black curve is corresponding to 80 mg/dL BGL while the blue one is representing the 124 mg/dL reading that leveled up two hours after the meal intake.

To better understand the BGL detection, the measured sensor data was further analyzed and processed using the discrete Fourier transform (DFT) algorithm. The consequent energy density has shown to be varying for the two processed data corresponding to the two different BGL readings, 80 and 124 mg/dL, as depicted in the enclosed plot in Fig. 13e, that shows an energy density metric of about 1,207 and 1,122 Volts^2^, respectively. This would also imply that the sensitivity to glucose variation is slightly reduced when compared to that of the samples in glassy container. In fact, the coupled electric field in the sensing region has less interaction with the glucose molecules in this case given the lossy nature of the fingertip biological structure including the cornified skin layer. This is seen very clearly when a skin layer model was introduced in the numerical simulations showing the field intensity with decaying magnitudes halfway through the glucose-contained layer. However, the sensitivity could be enhanced by modifying the design specifications through incorporating a flexible substrate of smaller loss tangent or utilizing a more powerful driving circuit (> 1 Watt output power) instead of the RDKIT used in this preliminary prototype as a proof-of-concept.

To confirm the correlation of the sensor readings to that of the actual BGL in real-time setting, another experiment was performed while continuously monitor a volunteer’s BGL over a course of 30 min before and after a meal. First, the pre-prandial test was conducted, and the corresponding sensor data were collected every 10 min resulting into four distinct readings. At each trial instant, the measurement was repeated for three times while placing the fingertip and the average of was plotted in Fig. 13f in terms of peak amplitudes (blue curve). The invasive readings were collected accordingly using the Glucometer and plotted in Fig. 13f (red curve). The sensor measurements follow the trend of the reference BGL that increases slightly in the range 93.7–101 mg/dL. In this narrow range of BGL variation, the sensor readings exhibited a repeatability error of about ± 0.0198 V max.

The post-prandial test was performed similarly right after the meal (~ 10 min) by collecting four distinct readings over a period of 30 min. The average of three repeatable sensing trials was plotted in Fig. 13g in terms of the peak amplitudes with a repeatability error of about ± 0.019 V. The invasive measurements revealed a significant jump to 165.7 mg/dL 10 min after the meal intake, then dropped slightly to 155, 146, then 10 mg/dL by the fourth check performed 40 min after the meal. The sensor readings follow this descending pattern as depicted in Fig. 13g. The sensor results for both pre- and post-prandial measurements shows no delay compared to the reference BGL, thus indicating the direct BGL monitoring from blood. The in-vivo measurements attained in this study have not investigated the effect of physical activities or the physiological differences between different subjects on the sensor responses; and that will be further explored in future studies on many patients with different diabetes conditions.

## Methods

### Sensor fabrication

The sensor prototype was fabricated on an FR4 PCB of a copper thickness of 35 *um* and dielectric substrate of 0*.*8 mm thickness using the laser technology incorporated in the proto-laser machine (LPKF ProtoLaser U4). First, the DipTracer PCB layout software was used to generate the Gerber files (.gbr) from the DXF HFSS design files for the CSRR structure. A few fiducial points were added to the design to ease the alignment of top and bottom parts in fabrication. The CircuitPro software was used to control the laser micromachining when fabricating the CSRR device. On the top layer of the PCB, three circular holes were created and drilled through the PCB using the scanning laser beam in order to perfectly align top and bottom parts using the integrated vision system and prefabricated alignment fiducials. Then the laser cutting was used to engrave the outer and inner dielectric annular slits in the four hexagonal-cells in the top layer of the PCB. Finally, the bottom metallic layer was patterned using the laser beam after alignment with fiducials by removing all the copper mass except an area of length 66 mm and width 1*.*5 mm that defines the MTL used for excitation purpose. Both ends of the MTL on the bottom surface of the structure were soldered to 50 Ω SMA coaxial connectors to ease the VNA connection for measurements.

### Fixture fabrication

The body of the fixture was made of acrylic, also known as “Poly Methyl Methacrylate”. The thicknesses of the material used to construct the overall body were 9 mm and 6 mm. The base of the fixture consists of 6 mm acrylic, which was cut using Epilog Fusion 40, a 75 W carbon laser cutter. The design and the thickness of the base plate allows the device to be stable when in use. The upper part of the body consists of 9 mm acrylic, designed to provide stability to the user’s fingertip when using the device. The thickness is particularly useful for allowing the users to precisely place their finger onto the sensor for accurate measurements. To precisely combine the upper part of the fixture with the lower part, there were a multitude of methods used. The first was the cut holes, that allow the both parts to be screwed together. Another key aspect to aid the placement of the parts was engraving the base of the acrylic about 2 mm deep so that the sensor could be accurately placed in between the acrylic plates. Overall, the design and the material used were professionally chosen to allow for both portability and precision for preliminary tests on the sensor.

### Preparation of samples

The tested samples of glucose aqueous solutions were prepared precisely in disparate glucose concentrations of 70*,* 90*,* and 110 mg/dL using a micropipette device in the laboratory. A standard glucose solution of high concentration *C*_1_ = 1,000 mg/dL (Carolina) was provided to save the preparation time and limit the possible errors in the prepared concentrations. The dilution equation *C*_1_*V*_1_ = *C*_2_*V*_2_ was used to calculate the volume *V*_1_ added from the stock solution to achieve the lower concentrations desired for the prepared glucose samples of *V*_2_ = 0*.*20 dL each. The measured stock quantity was then transferred using the micropipette and diluted with distilled water (DI). The desired concentrations of the prepared glucose samples were confirmed by testing a hemoglobin-mixed version of these samples on a reference glucometer device.

### Instruments and setups

Measurements of the scattering-parameters for the fabricated CSRR glucose sensors were accomplished using the Keysight Technologies 85,052 D PNA-L 2-port Vector Network Analyzer (VNA). The Open-Short-Load standard technique was used to calibrate the VNA in the frequency range of interest. The S-parameters data were recorded at a room temperature of 25 °C with a resolution of 800 frequency points and a test port of − 10 dB input power. The MIT coffee-can radar board was used to realize the portable setup of the sensor. It operates with the following specifications: 2.45 GHz centre frequency, 100 MHz max bandwidth, 16 bits ADC, 20,000 Hz ADC sampling rate, 5 V operating voltage, 0.5 Amp operating current, 1- and 0.125- Watt output power for the sweeping and CW modes, respectively. The glucose samples were tested inside a glassy container with  ${\epsilon }_{r}$ = 3.9 and *tanδ* = 0.001 and wall thickness of 1.5 mm. The total exposed volume of glucose samples was 600 µL. Simulations were carried out using the FEM-based High Frequency Structure Simulator (ANSYS HFSS) where the driven solution of each simulation model was set with the appropriate parameters of solution frequency, maximum number of passes, delta S for allowable variation between consecutive meshes, and minimum converged passes. The port and meshing sizes were also chosen suitably to achieve the convergence criteria accordingly. The invasive blood glucose measurements were collected using the iHealth commercial glucometer.

### Processing algorithms

The raw data of the CSRR sensor were processed using two techniques: (1) The energy density algorithm was implemented in MATLAB for analyzing the amplitude readings collected from the radar receiving channel. (2) The PCA algorithm was also executed in MATLAB to extract the feature vectors from the transmission responses as recorded by the VNA and RDKIT then applying the classification routine to depict the principal components for tested glucose samples in 2D plots.

## Conclusion

A low-cost microwave sensor of high sensitivity was presented for monitoring the blood glucose levels of diabetes. The microwave sensor was realized using a microstrip structure with an improved CSRRs configuration of a honey-cell fashion. Two preliminary prototypes were numerically modelled, fabricated and experimented for monitoring the glucose level changes of 70–120 mg/dL in blood mimicking aqueous solutions. The proposed CSRR sensor has exhibited an impressive detection sensitivity for the glucose-level variations in the measurements collected using two different setups of an expensive VNA and a low-cost low-frequency radar. Particularly, the compact topology of the sensor has shown distinguishable frequency shifts of about 0.94 MHz/(mg/dL) resolution at several transmission resonances in response to the varying-level glucose samples. The detection capability of the changes in the scattering responses of the sensor were further enhanced by applying the PCA feature extraction algorithm to clearly identify blood samples of different concentrations. Additionally, a preliminary in-vivo experiment has demonstrated the efficacy of the integrated sensor in tracking the blood glucose level patterns from the fingertip. With appropriate AI-powered signal processing and a one-time personalized invasive calibration, the proposed device promotes the potentiality of personalized, fast, accurate, and non-invasive monitoring of the blood glucose level for diabetes control and prevention. Moreover, the sensor miniature scale offers a great advantage for realizing it as a wearable technology (e.g. smart watch) for continuous glucose sensing similar to those of breath and heart rates. Results of this study could be considered as a paradigm shift in microwave sensors for personalized biomedical-specific applications like diabetes monitoring and pave the way towards their commercialization.

### Ethical approval

The authors confirm that all the methods used in the in-vitro and in-vivo experiments were carried out in accordance to the relevant guidelines and regulations at University of Waterloo.
